# MoaB2, a newly identified transcription factor, binds to σ^A^ in *Mycobacterium smegmatis*

**DOI:** 10.1128/jb.00066-24

**Published:** 2024-11-05

**Authors:** Barbora Brezovská, Subhash Narasimhan, Michaela Šiková, Hana Šanderová, Tomáš Kovaľ, Nabajyoti Borah, Mahmoud Shoman, Debora Pospíšilová, Viola Vaňková Hausnerová, Dávid Tužinčin, Martin Černý, Jan Komárek, Martina Janoušková, Milada Kambová, Petr Halada, Alena Křenková, Martin Hubálek, Mária Trundová, Jan Dohnálek, Jarmila Hnilicová, Lukáš Žídek, Libor Krásný

**Affiliations:** 1 Laboratory of Microbial Genetics and Gene Expression, Institute of Microbiology of the Czech Academy of Sciences, Prague, Czechia; 2 Central European Institute of Technology (CEITEC), Masaryk University, Brno, Czechia; 3 Faculty of Science, National Centre for Biomolecular Research, Masaryk University, Brno, Czechia; 4 Institute of Biotechnology of the Czech Academy of Sciences, Centre BIOCEV, Vestec, Czechia; 5 Laboratory of Regulatory RNAs, Faculty of Science, Charles University, Prague, Czechia; 6 Institute of Microbiology of the Czech Academy of Sciences, Centre BIOCEV, Vestec, Czechia; 7 Institute of Organic Chemistry and Biochemistry, Czech Academy of Sciences, Prague, Czechia; The Ohio State University, Columbus, Ohio, USA

**Keywords:** MoaB2, σ^A^, mycobacteria, RNA polymerase, transcription

## Abstract

In mycobacteria, σ^A^ is the primary sigma factor. This essential protein binds to RNA polymerase (RNAP) and mediates transcription initiation of housekeeping genes. Our knowledge about this factor in mycobacteria is limited. Here, we performed an unbiased search for interacting partners of *Mycobacterium smegmatis* σ^A^. The search revealed a number of proteins; prominent among them was MoaB2. The σ^A^-MoaB2 interaction was validated and characterized by several approaches, revealing that it likely does not require RNAP and is specific, as alternative σ factors (*e.g.*, closely related σ^B^) do not interact with MoaB2. The structure of MoaB2 was solved by X-ray crystallography. By immunoprecipitation and nuclear magnetic resonance, the unique, unstructured N-terminal domain of σ^A^ was identified to play a role in the σ^A^-MoaB2 interaction. Functional experiments then showed that MoaB2 inhibits σ^A^-dependent (but not σ^B^-dependent) transcription and may increase the stability of σ^A^ in the cell. We propose that MoaB2, by sequestering σ^A^, has a potential to modulate gene expression. In summary, this study has uncovered a new binding partner of mycobacterial σ^A^, paving the way for future investigation of this phenomenon.

**IMPORTANCE:**

Mycobacteria cause serious human diseases such as tuberculosis and leprosy. The mycobacterial transcription machinery is unique, containing transcription factors such as RbpA, CarD, and the RNA polymerase (RNAP) core-interacting small RNA Ms1. Here, we extend our knowledge of the mycobacterial transcription apparatus by identifying MoaB2 as an interacting partner of σ^A^, the primary sigma factor, and characterize its effects on transcription and σ^A^ stability. This information expands our knowledge of interacting partners of subunits of mycobacterial RNAP, providing opportunities for future development of antimycobacterial compounds.

## INTRODUCTION

Mycobacteria are medically important Actinobacteria that contain human pathogens such as *Mycobacterium tuberculosis*, *Mycobacterium leprae*, and *Mycobacterium abscessus. Mycobacterium* (synonym *Mycolicibacterium) smegmatis* is a fast-growing, non-pathogenic relative of these species (1, 2). Interestingly, it still contains a number of genes recognized as important for virulence in *M. tuberculosis* (3). More importantly, *M. smegmatis* and *M. tuberculosis* display high similarity in the composition of the transcription machinery, which mediates the first step of gene expression, a process essential for the functioning of the cell and its adaptation to changing environment.

In bacteria, the central enzyme of transcription is multisubunit RNA polymerase (RNAP). The RNAP core consists of several subunits (α_2_ββ'ω) (4). This core is capable of transcription elongation and termination but not initiation. To initiate, the RNAP core needs to bind a sigma factor (σ) to form the holoenzyme (5). This holoenzyme recognizes specific DNA sequences called promoters from which transcription initiates. Bacterial species typically contain various σ factors that provide specificity for different promoter sequences. Prominent among these factors is the vegetative (primary) σ, σ^A^ (σ^70^ in *Escherichia coli*) that is active mainly during the exponential phase of growth and drives transcription of housekeeping genes. The other σ factors are usually referred to as alternative σ factors.

Activities of alternative σ factors are regulated by anti-σ factors (6). They bind to σ factors and block their binding to RNAP. Anti-σ factors consist of a σ-binding domain and a signaling domain that responds to signals from the inside or outside of the cell. They are poorly conserved at the sequence level and are often co-transcribed with their respective σ factor genes (5). Primary σ factors typically do not have counterpart anti-sigma factors, although the Rsd protein of *E. coli* was shown to bind to σ^70^ and modulate gene expression (7).

*M. smegmatis* contains 28 σ factors (8, 9), most of which are still poorly characterized. The primary σ factor, σ^A^, consists of four domains (σ^A^_N_, σ^A^_2_, σ^A^_3_, and σ^A^_4_). Both *M. smegmatis* and *M. tuberculosis* contain an alternative σ factor, σ^B^, 64% identical with σ^A^ (amino acid identity) that directs transcription of genes expressed in stationary phase and during stress response and was also proposed to induce oligomerization of RNAP, capturing it in an inactive conformation (10–14). Moreover, recently published work characterizing *M. smegmatis* σ^B^ identified its involvement also during exponential phase where σ^B^ binds to >200 promoter regions, including those driving expression of essential housekeeping genes (15). σ^B^, though, is not essential and differs from σ^A^ by the absence of the N-terminal domain.

The *E. coli* σ^70^ N-terminal domain is called 1.1 and has a close-packed folded structure (16). This structure is found in most bacterial species (17, 18). To the contrary, in Actinobacteria including *M. smegmatis*, the N-terminal regions of primary σ factors display divergent primary amino acid sequences and are predicted to be unfolded. Hence, this domain in mycobacteria is not termed σ^A^_1.1_ but σ^A^_N_ (19, 20). Functionally, it was shown to be important for transcription *in vitro* and its absence negatively affected survival (21). The exact mechanistic functioning and structure of this domain in Actinobacteria are not yet defined.

Previous studies of mycobacteria have yielded discoveries of general factors required for proper functioning of the transcription machinery, CarD and RbpA (22–29). These proteins bind to RNAP and affect the stability of the transcription bubble during initiation. Overall, however, our understanding of the mycobacterial transcription system is still lagging behind that of the model organism *E. coli*. Here, as part of our effort to extend our knowledge about mycobacterial transcription apparatus, we identify a new interacting partner of *M. smegmatis* σ^A^, MoaB2, determine its 3D structure, and show that it does not bind to alternative σ factors but requires the N-terminal domain of σ^A^ for the interaction. Subsequently, we demonstrate that while this protein is not essential, it modulates mycobacterial transcription *in vitro* and may affect stability of σ^A^ *in vivo*.

## RESULTS

### σ^A^ binds/is in a complex with MoaB2

To start characterizing σ^A^ and the complexes it is present in, we used laboratory wild-type (wt) strain *M. smegmatis* mc^2^ 155 and fused the *mysA* gene (*MSMEG_2758*) that encodes σ^A^ in its native locus with the sequence encoding the FLAG epitope. The affinity tag was positioned at the C-terminus of σ^A^ (σ^A^-FLAG). Next, using the created σ^A^-FLAG strain and the parent wt strain without any FLAG (no FLAG strain as a negative control), we pulled down proteins by immunoprecipitation (IP) from exponential and stationary phases of growth with a monoclonal antibody recognizing the FLAG epitope. The immunoprecipitated proteins were digested in solution and analyzed by liquid chromatography coupled with mass spectrometry (LC-MS) that allows the characterization of complex protein mixtures with high sensitivity.

**Fig 1:**
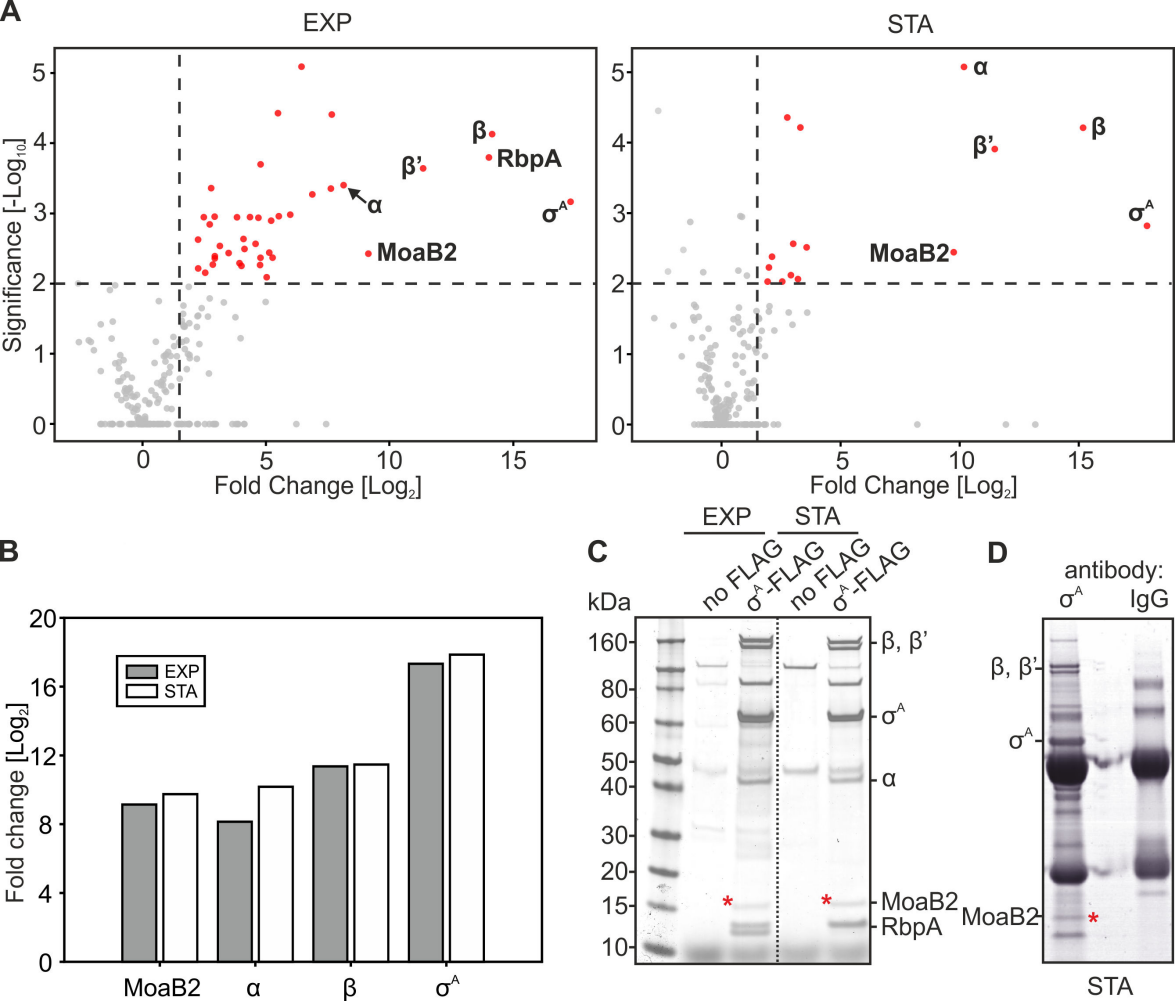
*M. smegmatis* MoaB2 is in the interactome of σ^A^. (**A**) Volcano plots of proteins associating with *M. smegmatis* σ^A^-FLAG (strain LK3207) pulled down in exponential (EXP) and stationary (STA) phases of growth. The plots show LC-MS-identified proteins enriched in IP pull downs with anti-FLAG over proteins from the control “no FLAG” strain (LK3016). Red spots indicate proteins significantly enriched (−log_10_ *P* < 2, indicated with the horizontal dashed line; enrichment >log_2_>2, indicated with the vertical dashed line). The spots show averages from three independent biological repeats. (**B**) Quantitation of relative enrichments of selected σ^A^-FLAG (LK3207) associating proteins from (**A**) compared to the “no FLAG” strain. Data from exponential (EXP) and stationary (STA) phases are indicated. The bars show averages from three independent biological repeats. The SDs cannot be shown directly in the graph because they are calculated from the intensity values, whereas the fold change is shown in the graph. However, the variance of the replicates is one of the parameters of the *P*-value calculated in the *t*-test—the lower the variance, the lower the *P*-value (*P*-value_MoaB2_STA_ = 0.0036; *P*-value_MoaB2_EXP_ = 0.0037; *P*-value_α_STA_ < 0.001; *P*-value_α_EXP_ < 0.001; *P*-value_β_STA_ < 0.001; *P*-value_β_EXP_ < 0.001; *P*-value_σA_STA_ = 0.0015; *P*-value_σA_EXP_ < 0.001). (**C**) SDS-PAGE of IPs of FLAG-tagged σ^A^ (LK3207) using the anti-FLAG antibody. “No FLAG” strain is negative control (LK3016). Data from exponential (EXP) and stationary (STA) phases are shown. The dotted line indicates that this panel was electronically assembled from two parts of one gel. MoaB2 is marked with red asterisk. The identity of the bands was determined by mass spectrometry. The experiment was performed in three biological replicates [independent of experiments shown in (A)] with the same result. (**D**) SDS-PAGE of IP of σ^A^ from the stationary phase “no FLAG” strain cells (wt, LK3016) using antibody against σ^70^ (anti-σ^70^, clone name 2G10). IgG is a mouse nonspecific IgG used as a negative control. The band corresponding to MoaB2 is indicated with red asterisk. The identity of the bands was determined by mass spectrometry. The experiment was performed in three biological replicates with the same result.

Figure 1A and B shows enrichment (threshold log_2_ = 2) of proteins pulled down from the strain containing σ^A^-FLAG over proteins pulled down from the control strain (non-specific binding). Pull-down from the exponential phase yielded a higher number of significantly enriched proteins than pull-down from stationary phase (Table S1). Unsurprisingly, in both growth phases, σ^A^ was the most enriched protein as well as core subunits of RNAP (α, β, and β’). RbpA, the transcription factor, was also highly enriched in exponential phase. Other enriched proteins included, *e.g*., MoaB2 (*MSMEG_5485*), F420 (*MSMEG_0777*), CbiA (*MSMEG_0067*), and Pks (*MSMEG_0408*). These proteins have various predicted functions that are not primarily associated with transcription (Table S1).

To identify the most prominent interacting partner(s), we repeated the IP experiments and analyzed the immunoprecipitated proteins on SDS-PAGE (Fig. 1C). Consistent with the previous results, a band of the size corresponding to MoaB2 was clearly visible in the gels. The MoaB2 identity was verified by mass spectrometry.

MoaB2 was found to be highly enriched in both growth phases (Fig. 1A through C). MoaB2 is a 17.9 kDa (pI 4.17) protein of unknown function. In *E. coli*, it has two homologs, MoaB (30% aa identity) and MogA (29% aa identity). MoaB was originally believed to be involved in the biosynthesis of molybdenum cofactor (Moco) (30) but subsequent genetic and biochemical experiments revealed that it does not play any role in this process (31, 32). MogA is involved in Moco biosynthesis (33).

To rule out the possibility that the σ^A^-MoaB2 interaction was not an artifact caused by the presence of the FLAG-tag on σ^A^, we used a different antibody, this time recognizing directly σ^A^ in the wt strain without any affinity tag (no FLAG). Using stationary phase cells, we observed that MoaB2 was found in complex with σ^A^ even in this genetic background (Fig. 1D).

The mass spectrometry analysis also revealed that the MoaB2 protein interacting with σ^A^ was 164 amino acids (aa) long and not 178 aa as annotated in Mycobrowser (34) (*MSMEG_5485*) and Uniprot (A0R3I5). Based on our data, the translation of MoaB2 starts with a methionine (encoded by ATG) 14 aa downstream of the annotated putative translation start (GTG; see Fig. S1).

We concluded that MoaB2 in the cell is shorter than the annotated version and binds σ^A^ either directly or in complex with RNAP.

### MoaB2 binds neither RNAP nor selected alternative σ factors

We next set out to determine whether MoaB2 binds to RNAP or interacts directly with σ^A^ in the absence of RNAP and whether MoaB2 also binds alternative σ factors. For this purpose, we used a *M. smegmatis* strain encoding a C-terminal FLAG-tag on the β subunit of RNAP (strain LK1468) and created strains with ectopically integrated anhydrotetracycline (ATC) inducible genes for selected σ factors (FLAG-tagged) as well as the same type of strain for σ^A^. This approach was used to circumvent the need for specific conditions associated with the activity of these σ factors. These factors were σ^B^, σ^E^, σ^F^, σ^G^, and σ^H^.

σ^E^ (*MSMEG_5072*) together with σ^B^ (*MSMEG_2752*) were suggested to play roles in transition to antibiotic-tolerant persistence and in situations when respiratory electron transport chain is inhibited (35, 36). σ^F^ (*MSMEG_1804*), phylogenetically and functionally similar to the general stress σ^B^ factor from *Bacillus subtilis* (37, 38), was implicated in adaptation to stationary phase and conditions of heat and oxidative stress as well as in carotenoid (isorenieratene) pigmentation associated with increased susceptibility to hydrogen peroxide (39–41). The function of σ^G^ (*MSMEG_0219*) is still undefined and σ^H^ (*MSMEG_1914*) regulates a transcriptional network that responds to heat and oxidative stress (42).

**Fig 2:**
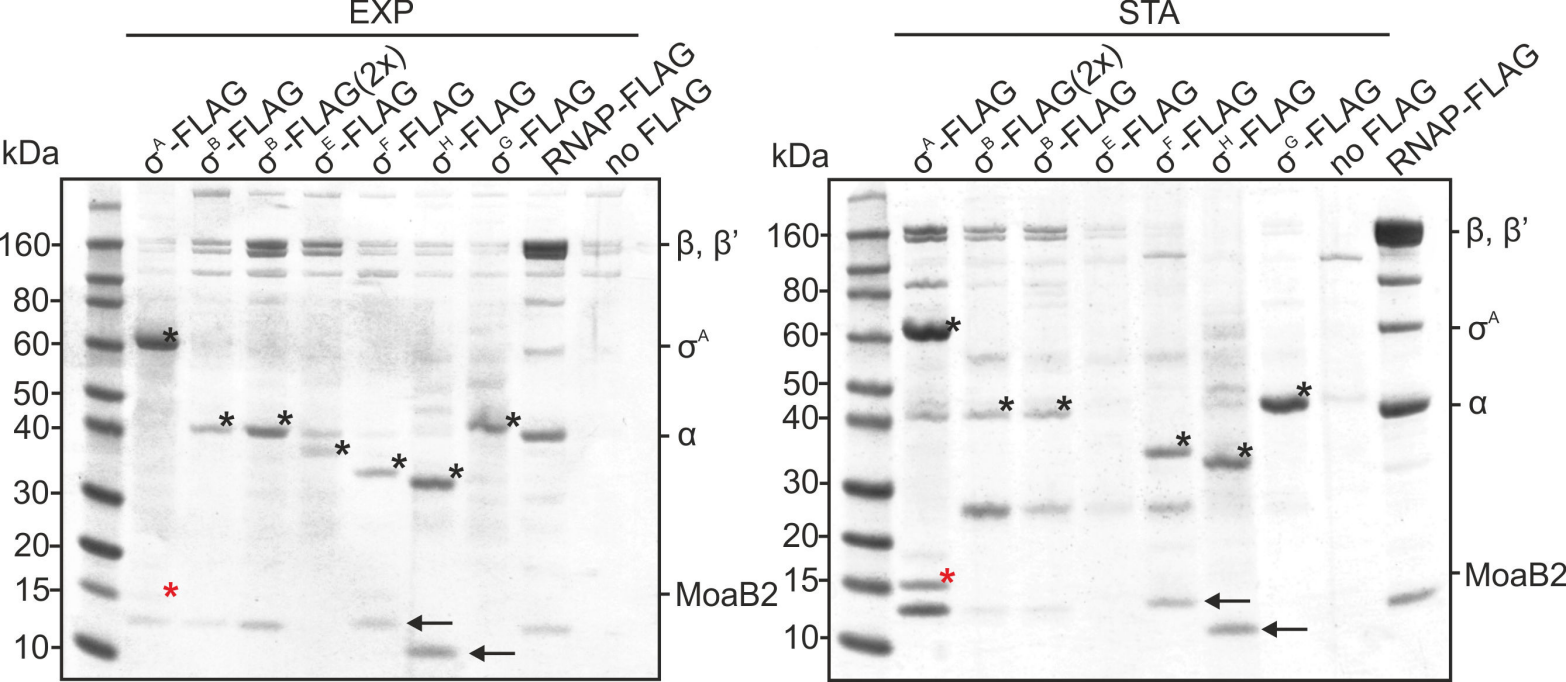
*M. smegmatis* MoaB2 does not associate with alternative σ factors. SDS-PAGE of IPs of FLAG-tagged sigma factors [σ^A^ (LK2073), σ^B^ (LK2077), σ^E^ (LK2157), σ^F^ (LK2159), σ^H^ (LK2160), σ^G^ (LK2161), or FLAG-tagged β´ subunit of RNAP (LK1468)] using anti-FLAG antibody. The FLAG-tagged proteins were present in the genome in an additional copy under ATC inducible promoter and expressed after ATC induction. In the case of σ^B^ (2×), twofold amount of cells were harvested for IP to enhance the detection of MoaB2 if present. The “No FLAG” strain was used as a negative control (LK3016). Individual σ factors are marked with black asterisks. Black arrows indicate respective anti-σ factors. MoaB2 is marked with red asterisk. The identity of the bands was determined by mass spectrometry. Three independent experiments were performed with identical results. Visualization of proteins was done also by silver staining with the same result (Fig. S2).

We performed IPs with anti-FLAG antibody from respective strains from exponential and stationary phases and analyzed the results on SDS-PAGE (Fig. 2; Fig. S2). First, the experiments revealed that MoaB2 was absent from a complex with RNAP that also contained σ^A^, strongly arguing that σ^A^ and MoaB2 do not interact in the context of RNAP, at least not in detectable amounts. Second, the experiments showed that no other tested alternative σ factor had pulled down MoaB2. An exception was σ^E^ in stationary phase where σ^E^ did not accumulate, likely being rapidly degraded. In this case, it was not possible to make a conclusion.

### σ^A^ and MoaB2 form a complex *in vitro*

**Fig 3:**
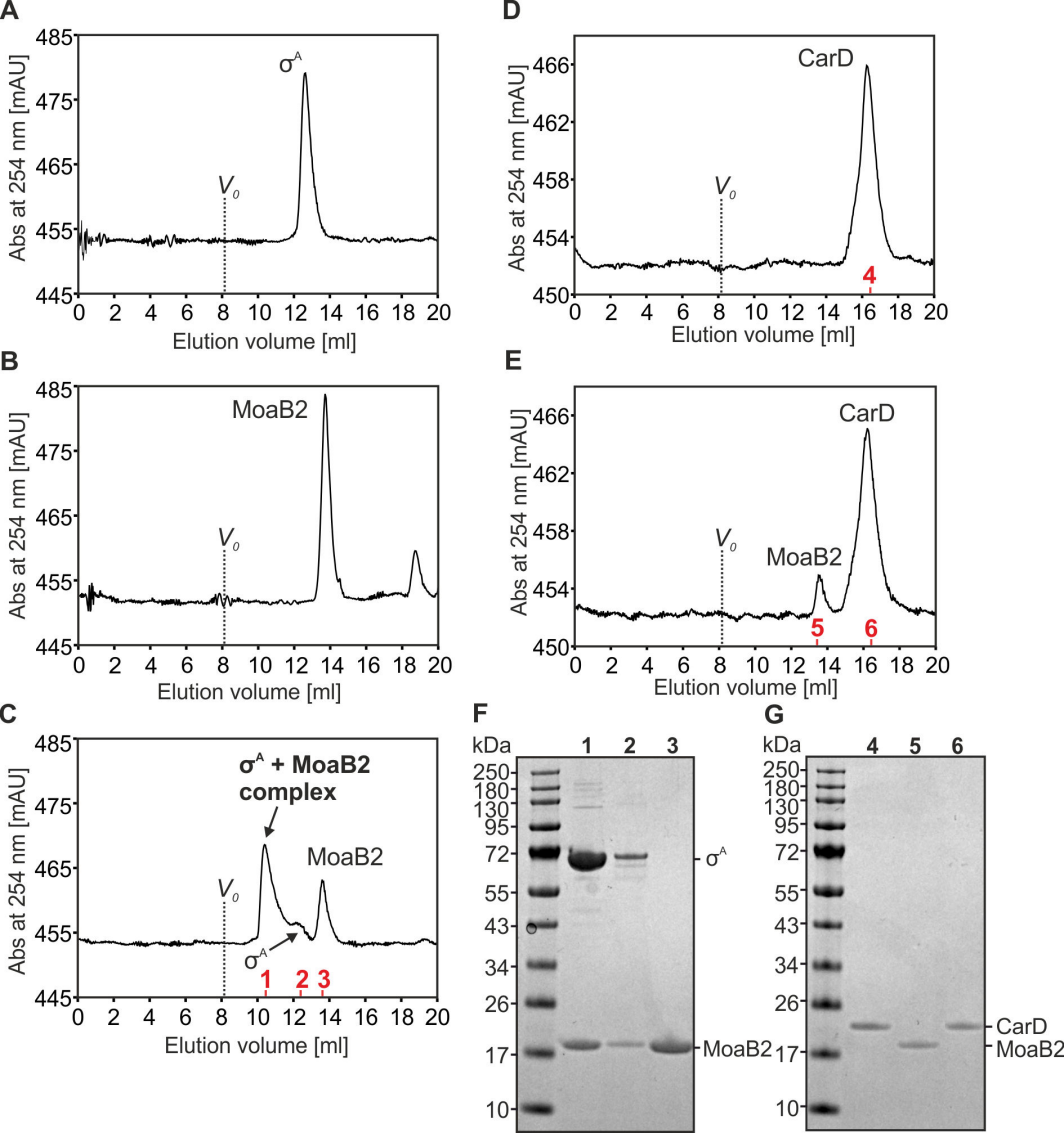
*M. smegmatis* σ^A^ interacts with *M. smegmatis* MoaB2 *in vitro*. All SEC runs were done using Superdex 200 Increase 10/300 Gl column (GE Healthcare) calibrated using Blue dextran and six protein standards ranging from 12.4 to 669 kDa selected from Gel Filtration Markers Kit for Protein Molecular Weights (MW) 6,500–66,000 Da (Sigma-Aldrich, MWGF70) and Gel Filtration Markers Kit for Protein Molecular Weights (MW) 29,000–700,000 Da (Sigma-Aldrich, MWGF1000). Void volume (***V_0_***) is marked. (**A**) SEC chromatogram of σ^A^ from *M. smegmatis*. Peak elution volume was 12.6 mL, which suggests MW of ~122 kDa. Fractions eluted at 12 to 13.5 mL were pooled and used for formation of the σ^A^-MoaB2 complex. (**B**) SEC chromatogram of MoaB2 from *M. smegmatis*. Peak elution volume was 13.6 mL, which suggests MW of roughly 77 kDa. Fractions eluted at 13 to 14.5 mL were pooled and used for formation of the σ^A^-MoaB2 complex. (**C**) SEC chromatogram of σ^A^-MoaB2 complex formed by mixing samples from (**A**) and (**B**). σ^A^ and MoaB2 were mixed at a molar ratio (monomer:monomer) of 1:2. Peak elution volume corresponding to σ^A^-MoaB2 complex was 10.4 mL, to unbound σ^A^ it was estimated to be 12.4 mL and to unbound MoaB2 13.6 mL. These elution volumes indicate MW of approximately 330 kDa,133 kDa, and 77 kDa, respectively. σ^A^ elutes before MoaB2 at a lower volume than expected according to its MW because it is a non-globular protein with long loops (the longest intramolecular distance in the structured part of σ^A^ is ~116 Å) and contains an intrinsically disordered N-terminal domain. The hexamer of MoaB2 is globular (the longest intramolecular distance ~80 Å). Individual peaks are marked with respective proteins, and corresponding fractions used for SDS-PAGE analysis (see Panel F) are indicated with red numbers. (**D**). SEC chromatogram of CarD from *M. smegmatis*. Peak elution volume was 16.3 mL that suggests MW of ~23 kDa. Fractions eluted at 16–16.5 mL were pooled and used in E to test whether MoaB2, under the used experimental conditions, does not form unphysiological complexes. The fraction used for SDS-PAGE analysis (see Panel G) is indicated with the red number. (**E**) SEC chromatogram of CarD and MoaB2 from *M. smegmatis*. Molar ratio of CarD and MoaB2 was approximately 1:1 (see Panel G). Complex between MoaB2 and CarD is not forming as there was no new peak present, and the amount of CarD eluting at the position of “free” CarD (16.3 mL) in both experiments is the same. Peak elution volume corresponding to MoaB2 was 13.5 mL which suggests MW of ~81 kDa. Individual peaks are marked with respective proteins, and corresponding fractions used for SDS-PAGE analysis (see Panel F) are indicated with red numbers. (**F**) SDS-PAGE analysis of the σ^A^-MoaB2 complex formation. Lines 1–3 contain selected fractions from (**C**). MoaB2 in line 2 is present due to the tailing of σ^A^-MoaB2 complex peak which therefore overlaps with the peak of free σ^A^ at the elution volume from which the fraction was taken for the SDS-PAGE analysis. Color Prestained Protein Standard, Broad Range (New England Biolabs) was used as a marker. The experiment was performed in three independent replicas with identical results. (**G**) SDS-PAGE analysis of the MoaB2-CarD. Lines 4–6 contain selected fractions from (**D**) and (**E**). SDS-PAGE was performed under same conditions as in Panel F.

Subsequently, we performed size exclusion chromatography (SEC) experiments to test the ability of MoaB2 to form a complex with σ^A^ *in vitro* to reveal whether MoaB2 can directly interact with σ^A^, or whether another unknown factor is perhaps necessary for the MoaB2-σ^A^ complex formation. Figure 3A and B show that σ^A^ eluted sooner than MoaB2 when migrating alone. When mixed, a third peak appeared, migrating faster than σ^A^ (Fig. 3C), indicative of a larger complex. SDS-PAGE then confirmed the presence of both proteins in the respective fraction (Fig. 3F). CarD, used as a negative control protein, showed no interaction with MoaB2 (Fig. 3D, E and G). Thus, MoaB2 directly binds σ^A^ without the need for an additional factor.

### Stoichiometry of the σ^A^:MoaB2 complex

Analytical ultracentrifugation was employed to determine the stoichiometry and characteristics of the σ^A^/MoaB2 interaction. Analysis of sedimentation velocity (SV) data (Fig. S3A) revealed distinct size distributions for σ^A^ and MoaB2 proteins. σ^A^ was present predominantly in the monomeric form (3.0 S peak), while the c(s) distribution of MoaB2 consisted of the major hexamer and a minor trimer peak (~4.8 S and 3.6 S). The frictional ratio of σ^A^ (f/f_0_ ~ 1.55) was higher than the typical values for compact, globular proteins and in agreement with the partially disordered σ^A^ structure. The addition of MoaB2 to the σ^A^ sample led to a decrease in the σ^A^ monomer in solution and emergence of a new (complex) peak. When σ^A^ was in molar excess, the s-value of the complex was ~10 S. At higher MoaB2 concentrations, the distribution shifted to lower sedimentation coefficients, revealing the formation of smaller complexes with incomplete saturation by σ^A^.

Stoichiometry of the interaction was determined from the dependence of the peak area of the σ^A^ monomer on the increasing MoaB2:σ^A^ molar ratio. The analysis demonstrated a linear dependence of the peak area of free σ^A^ on the MoaB2:σ^A^ molar ratio for values below 0.75, confirming an overall 1:1 (σ^A^:MoaB2) binding stoichiometry (Fig. S3B). This was consistent across experiments, underscoring high reproducibility. The binding affinity was relatively high, with a dissociation constant much lower than the σ^A^ concentration used (~20 µM), in the submicromolar range (Fig. S3B).

To confirm these results, a multi-signal sedimentation velocity (MSSV) analysis (43) of the molar ratio 1:0.3 of σ^A^:MoaB2 was performed (Fig. S4). This procedure allowed us to determine the relative stoichiometry of the interacting molecules in the complex (provided that their spectral properties are sufficiently different). Here, we analyzed data obtained at 280 nm (where only σ^A^ absorbed) and using interference optics (where both proteins are detectable). The integration of the ~10 S complex peak after spectral decomposition gave an almost identical concentration of both components (5.62 µM σ^A^, 4.45 µM MoaB2), supporting the overall 1:1 stoichiometry.

### Determination of crystal structure of *M. smegmatis* MoaB2

**Fig 4:**
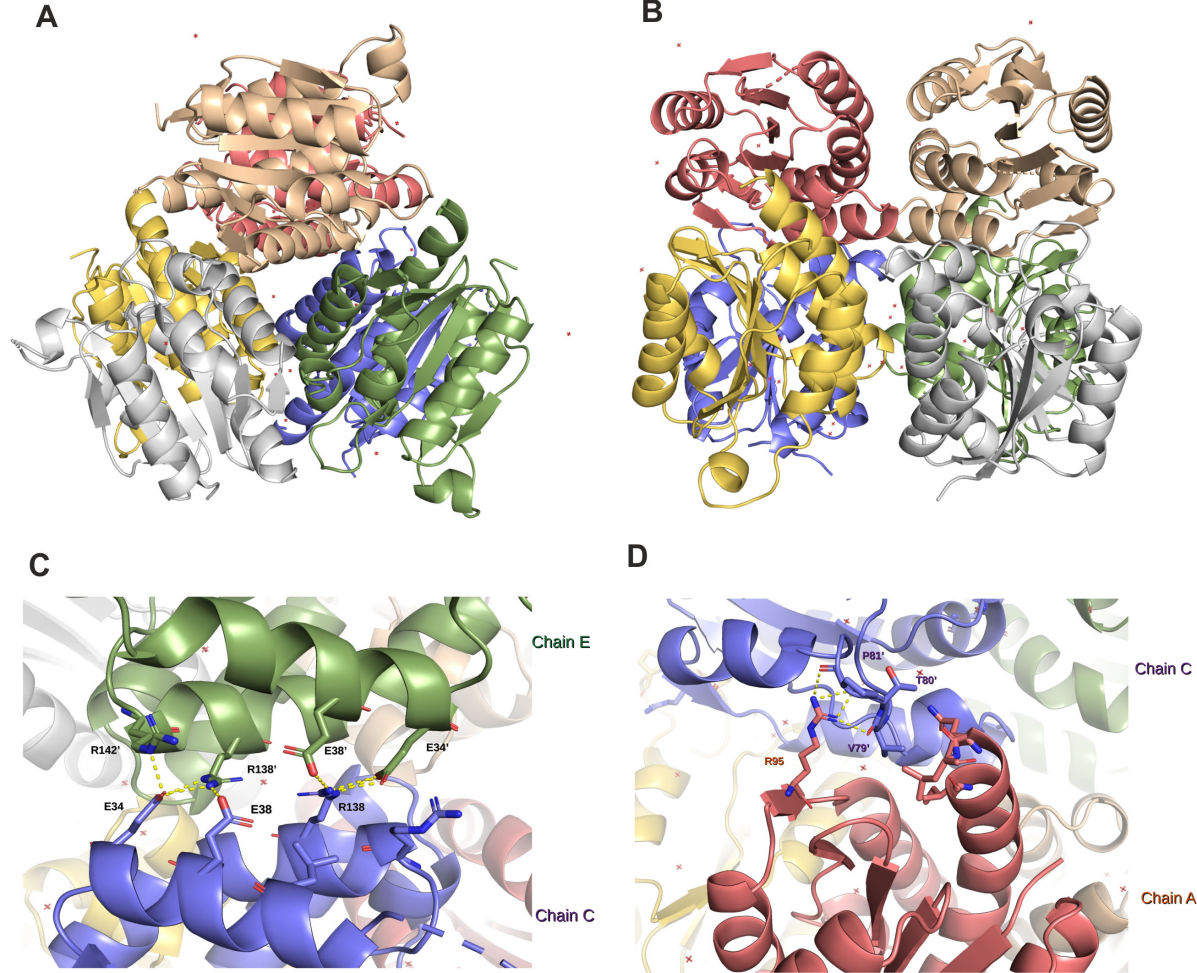
3D structure of *M. smegmatis* MoaB2. (**A**) Top view of the MoaB2 hexamer with the subunits shown in different colors. (**B**) Side view of the MoaB2 hexamer colored as in Panel A. (**C**) Detail of the interactions between monomeric subunits in the MoaB2 trimer. The hydrogen bond contacts at the interface (residues Val79, Thr80, and Pro81 from chain C and Arg95 from chain A) are shown, and the distances are specified in Å. (**D**) Detail of the trimer-trimer interface of MoaB2. The hydrogen bond and salt bridge contacts at the trimeric interface between Glu34, Glu38, Arg138, and Arg142 from chain E and chain C are shown.

To characterize *M. smegmatis* MoaB2 structurally, we cloned, overexpressed, and purified MoaB2. MoaB2 crystals were screened for diffraction, and diffraction data were collected (Table S2). The structure of *M. smegmatis* MoaB2 was solved by molecular replacement using *Mycobacterium marinum* MoaB2 (PDB id: 3rfq; sequence identity of 86% with *M. smegmatis* MoaB2) as a search model (44) (Fig. 4A and B). In the crystal, MoaB2 was present as a hexamer. The final crystallographic model was refined to an *R*_work_ of 0.192 (*R*_free_ 0.260). The data collection and refinement statistics from the final coordinates are summarized in Supplementary Table (Table S2). Each subunit of the hexamer exhibited a weak or missing electron density for the N-terminal residues 1–7, the C-terminal His-tag, and linker residues from the plasmid (165–172). Therefore, the mentioned residues could not be built properly.

The structure of each subunit of *M. smegmatis* MoaB2 is composed of a central β-sheet that is slightly curved and is surrounded by six α-helices and one 3_10_ helix. The electron density of chains A and F is poor, and thus, the 3_10_ helix is not visible. The β-sheet is made up of five parallel strands (β1–β4 and β6) and one antiparallel strand (β5), which are located between the inner β4 strand and the adjacent β6 strand (Fig. S5). The overall architecture of the protein subunit is similar to other known molybdopterin (MPT)-adenylyl-transferase enzymes.

The hexamers are made up of two trimers (chains ABC and DEF) making contacts through mostly hydrophobic and salt-bridge interactions at the interface between the subunits. Assembly of subunits into trimers within the hexamer is strengthened through several hydrogen bonds between Arg95 and Arg120 of one monomer and Val79, Thr80, and Pro81 of another monomer (Fig. 4C). Interactions between the two trimers (trimer ABC and trimer DEF) within the hexamer are mainly mediated by residues of α4- and α7-helices and the turn residues following α4-helix. Residues Leu31, Glu34, and Glu38 in α4-helix and Arg138 and Arg142 in α7-helix form the central core of the trimer-trimer interface (Fig. 4D). The residues forming the trimer-trimer interface are not conserved in all bacteria. Interestingly, the bacterial homolog MogA exists as a trimer both in crystal and solution. The eukaryotic orthologs, G-domain of Cnx1 from plants (Cnx1G), and G-domain of the multifunctional mammalian protein gephyrin were also shown to be trimers (45).

In crystal, the *M. smegmatis* MoaB2 hexamer forms the asymmetric unit. This differs from other MoaB structures (including its closest *M. marinum* homolog used as the search model in this study) with dimers or trimers in the asymmetric unit and forming hexamers through crystallographic symmetry. Nevertheless, all bacterial MoaB proteins are hexamers in solution (30, 46). SEC (Fig. 3B) and dynamic light-scattering experiments (Fig. S6) showed that *M. smegmatis* MoaB2 is a hexamer in solution as well. Calculations using the Adaptive Poisson-Boltzmann Solver (47) showed that the electrostatic potential on the surface of *M. smegmatis* MoaB2 is prominently negative (Fig. S7) (32, 48).

Structural similarity search using the DALI server (49) performed on MoaB2 then revealed a high level of homology with several proteins involved in Moco synthesis. The highest level of structural homology was observed with MoaB from *M. marinum* (PDB: 3rfq, Z-score of 32.9; rmsd of 0.4 Å over 153 Cα atoms). Among other homologs than MoaB, Molybdenum cofactor biosynthesis protein (MogA) from *Mycobacterium ulcerans* (PDB: 4twg, Z-score of 26.7; rmsd of 1.3 Å over 149 Cα atoms), Cnx1G (PDB: 1uux, Z-score of 26.1; rmsd of 1.3 Å over 152 Cα atoms from *Arabidopsis thaliana*) (50), G domain of gephyrin (PDB:1jlj, Z-score of 25.4; rmsd of 1.4 Å over 152 Cα atoms from *Homo sapiens*) (45), and the largest domain of MoeA (PDB: 1g8l, Z-score of 10.9; rmsd of 2.9 Å over 133 Cα atoms from *E. coli*) (51) showed closest similarities.

### σ^A^_N_ is important for the σ^A^-MoaB2 complex: interaction in the cell

**Fig 5:**
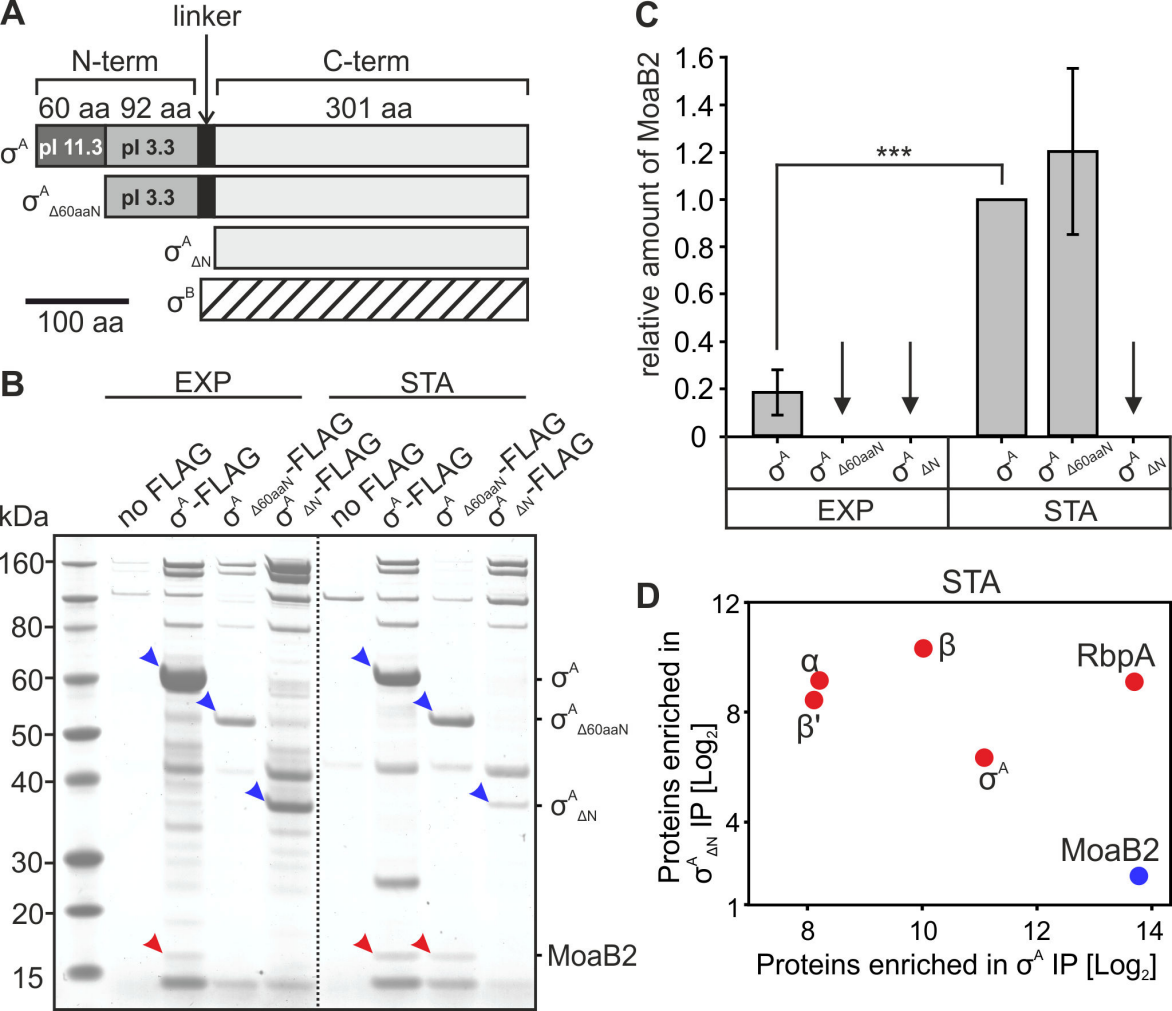
*M. smegmatis* σ^A^_N_ may be involved in the σ^A^-MoaB2 interaction. (**A**) Schematic linear representation of σ^A^, σ^A^_Δ60aaN_, σ^A^_ΔN_, and σ^B^. The scale bar represents 100 amino acids (aa). σ^A^ is 466 aa long, σ^A^_Δ60aaN_ is 401 aa long, σ^A^_ΔN_ is 301 aa long, and σ^B^ is 319 aa long. (**B**). SDS-PAGE gel of IP of FLAG-tagged proteins σ^A^ (LK2073), σ^A^_Δ60aaN_ (LK4207), and σ^A^_ΔN_ (LK2463) using anti-FLAG antibody. The dotted line indicates where this panel was electronically assembled from two parts of one gel. Relevant proteins are indicated on the right side of the gel; blue arrows mark different σ^A^ variants; red arrows indicate MoaB2. No FLAG strain (LK3016) was used as negative control. The identity of the bands was determined by mass spectrometry. The experiment was performed in three biological replicates with identical results. EXP, exponential; STA, stationary phase. (**C**) Relative amounts of *M. smegmatis* MoaB2 bound to different constructs of σ^A^ in EXP and STA phase of growth, calculated from signal intensities of respective bands from three independent SDS-PAGE gels from three independent experiments. QuantityOne (Bio-Rad) software was used for quantification. The relative values were normalized to molecular weight of proteins. The relative amount of σ^A^ immunoprecipitated from stationary phase (STA) was set as 1. The bars show the average from three biological replicates, and the error bars show ±SD. *P*-values that are less than 0.001 are marked as *** (*t*-test). The vertical arrows in the chart indicate values “zero” as the values were even below background. (**D**) Comparisons of proteins associating with σ^A^-FLAG (LK2073 over control LK3016) vs proteins associating with σ^A^_ΔN_-FLAG (LK2463 over control LK3016) as determined by quantitative LC-MS. Data from stationary phase (STA) are shown. Red spots indicate proteins significantly enriched (significance: −log_10_ *P* < 2; enrichment: >log_2_>2). The blue spot indicates MoaB2 that was not significantly enriched in σ^A^_ΔN_-FLAG (in σ^A^-FLAG it was significant). Each spot is the average calculated from three independent experiments. For values, see Supplementary Table (Table S3).

A major difference between σ^A^ and σ^B^ (as well as the other σ factors) is the absence of the N-terminal domain found in σ^A^ (σ^A^_N_) from σ^B^ (52) (Fig. 5A). Because we did not detect any binding of MoaB2 to σ^B^ (Fig. 2), we speculated that σ^A^_N_ may play a role in the interaction with MoaB2. Moreover, the negative charge of the surface of mycobacterial MoaB2 as revealed by the 3D structure suggested its potential interaction with the charged σ^A^_N_, especially with the 60 N-terminal positively charged aa.

To test the importance of the N-terminal domain of σ^A^ for the interaction with MoaB2, we created two strains with ectopically integrated ATC inducible variants of σ^A^ lacking σ^A^_N_ (σ^A^_ΔN_-FLAG) or σ^A^_N_ lacking the 60 N-terminal aa (σ^A^_Δ60aaN_-FLAG). IP experiments with these and control strains analyzed on SDS-PAGE showed that in exponential phase, only full-length σ^A^ was able to pull-down MoaB2 (Fig. 5B). In stationary phase, both full-length σ^A^ and σ^A^_Δ60aaN_ pulled down MoaB2, whereas σ^A^_ΔN_ was unable to do so (Fig. 5B). The relative amount of MoaB2 pulled down with σ^A^ was higher in stationary than in exponential phase (Fig. 5C).

To verify the absence of the interaction between σ^A^_ΔN_ and MoaB2 in stationary phase, we performed another set of pull-down experiments with strains containing full-length σ^A^ and σ^A^_ΔN_ (Table S3). The pulled-down proteins were digested in solution and analyzed by LC-MS. Figure 5D shows high and statistically significant enrichment of MoaB2 with full-length σ^A^ but not σ^A^_ΔN_.

Collectively, these experiments suggested that σ^A^_N_ is involved in the interaction between σ^A^ and MoaB2. Moreover, the 60 N-terminal aa do not seem to be essential for this interaction. Rather, the 61–160 aa region likely plays a role.

### σ^A^_N_ is important for the σ^A^-MoaB2 complex: NMR analysis

**Fig 6:**
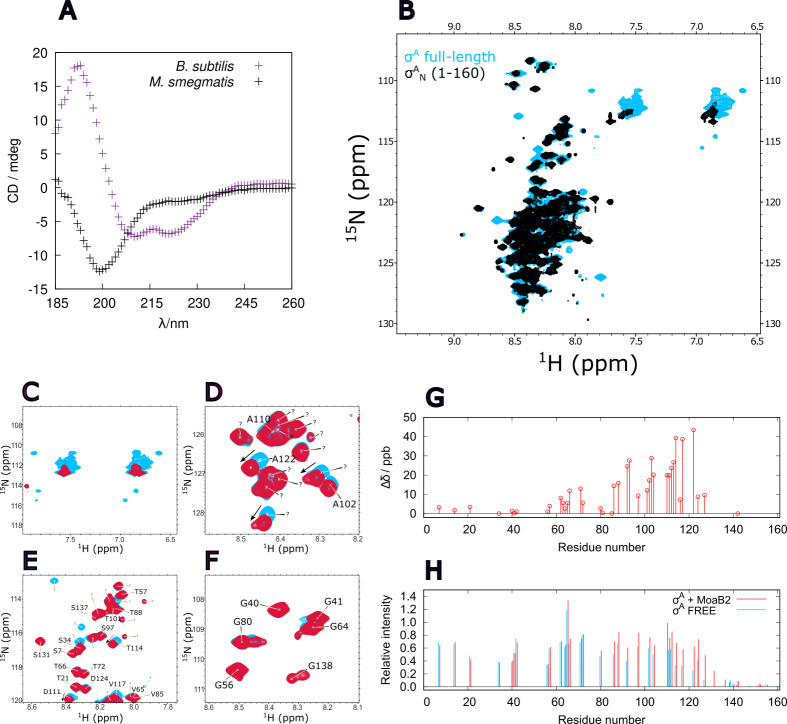
CD and ^1^H-^15^N HSQC NMR spectra of σ^A^ and in of σ^A^ with MoaB2. (**A**) CD spectra of σ_1.1_ from *B. subtilis* and σ^A^_N_ from *M. smegmatis*. The spectrum of *B. subtilis* σ_1.1_ domain (residues 1–72 of σ_1.1_ and additional eight residues of a His-tag) manifests a shape that is typical for well-ordered protein with high α-helical content, whereas the shape of *M. smegmatis* σ^A^_N_ (residues 1–165 of σ^A^_N_ preceded by a glycine) spectrum reveals a mostly disordered protein. (**B**) ^1^H-^15^N HSQC NMR spectra of *M. smegmatis* σ^A^_N_ (black contours) and full-length σ^A^ (light blue contours). The narrow dispersion of proton chemical shifts indicates a disordered protein since most of the peaks are clustered around the center of the measured spectrum and is not dispersed as is typical for proteins with well-defined structure. (**C–F**) Selected regions of ^1^H-^15^N HSQC NMR spectra of *M. smegmatis* full-length σ^A^. Spectra of free σ^A^ and σ^A^ with MoaB2 are shown in light blue and red, respectively. Peaks are labeled with single-letter symbols and residue numbers. (**G**) Plot of the combined chemical shift changes upon MoaB2 addition. (**H**) Plot of the relative heights of peaks of free σ^A^ (blue) and σ^A^ with MoaB2 (red).

As mycobacterial σ^A^_N_ had been predicted to be unstructured (19) (Fig. S8), we decided to test this prediction experimentally with purified protein samples *in vitro*. First, the disordered nature of σ^A^_N_ was confirmed by circular dichroism (CD) measurement, yielding a spectrum typical for random coil conformation, substantially different from a spectrum of the mostly helical domain 1.1 of σ^A^ from *B. subtilis* (Fig. 6A). Next, the ^1^H-^15^N HSQC NMR spectrum of σ^A^_N_ (residues 1–160) was acquired. The low dispersion of proton chemical shifts of backbone amides in the recorded spectrum (Table S4) confirmed that σ^A^_N_ adopts a disordered conformation (black contours in Fig. 6B).

To explore the importance of σ^A^_N_ for the σ^A^-MoaB2 interaction, we recorded ^1^H-^15^N HSQC NMR spectra (53, 54) of σ^A^_N_ (residues 1–160) alone and of σ^A^_N_ (residues 1–160) in the presence of MoaB2 (twofold molar excess). No significant difference between the spectra was observed (data not shown). The fact that the chemical shifts of σ^A^_N_ were not affected by the presence of MoaB2 indicates that σ^A^_N_ alone does not form a sufficiently strong complex with MoaB2.

Therefore, as the next step, ^1^H-^15^N HSQC NMR spectra of free full-length σ^A^ and of full-length σ^A^ in the presence of MoaB2 were recorded (blue contours in Fig. 6B). Most peaks in the spectrum of free σ^A^ corresponded to peaks of isolated σ^A^_N_ (consisting of amino acids 1–160), indicating that the majority of σ^A^ forms a folded structure of a size undetected by the ^1^H-^15^N HSQC experiment. Among several additional peaks, six corresponded to three amides of three side-chains of Asn or Gln outside σ^A^_N_, with chemical shifts of side-chain amides in well-ordered proteins. The peaks of the σ^A^_N_ were sharp as expected for disordered residues, with the exception of approximately 30 highly conserved residues of the C-terminal region of the σ^A^_N_.

As σ^A^ was not stable in solution at higher concentrations, the sample containing only the σ^A^_N_ was used to assign the σ^A^ peaks. Combining 5D HabCabCONH, triple resonance, and ^15^N-edited TOCSY and NOESY experiments, we obtained the assignment of 42% non-proline residues.

The addition of MoaB2 to σ^A^ changed several features of the spectrum: (i) peaks of side-chain amides outside the σ^A^_N_ disappeared (Fig. 6C), indicating the formation of a larger rigid structure; (ii) specific shifts of peak positions were observed (labeled by arrows in Fig. 6D and E, summarized in Fig. 6G), documenting a specific effect of MoaB2 on the corresponding residues; and (iii) heights of peaks in the C-terminal half of the σ^A^_N_ increased, most significantly for residues Glu111–Asp143 (*e.g*., peak S131 in Fig. 6E and peak G138 in Fig. 6F, summarized in Fig. 6H), suggesting that these residues became more flexible in the presence of MoaB2.

The fact that the addition of MoaB2 did not result in a dramatic peak broadening shows that MoaB2 does not bind tightly to σ^A^_N_. On the other hand, the specific peak shifts document that the σ^A^_N_ and MoaB2 interact. In agreement with results from the previous section, no peak of the N-terminal positively charged 60 amino-acid region is influenced by the strongly negatively charged MoaB2 protein. A combined chemical shift change higher than 0.02 ppm was observed for residues between Leu92 and Ala123. The highest values were observed in the _112_AATPAVATAKAA_123_ stretch. Finally, a comparison of peak broadening in the C-terminal region of the σ^A^ (not observed in the isolated free σ^A^_N_) suggests that this region interacts with the folded domains 2, 3, and 4 of σ^A^. In the presence of MoaB2, this interaction is partially released and limited to residues 145–160 that show predicted helical propensity.

Taken together, both the results from NMR and pull-down experiments point to a genuine interaction event and provide additional details about the interaction between MoaB2 and σ^A^, narrowing down the interaction region to the C-terminal part of σ^A^_N_.

### MoaB2 is not essential *in vivo*

**Fig 7:**
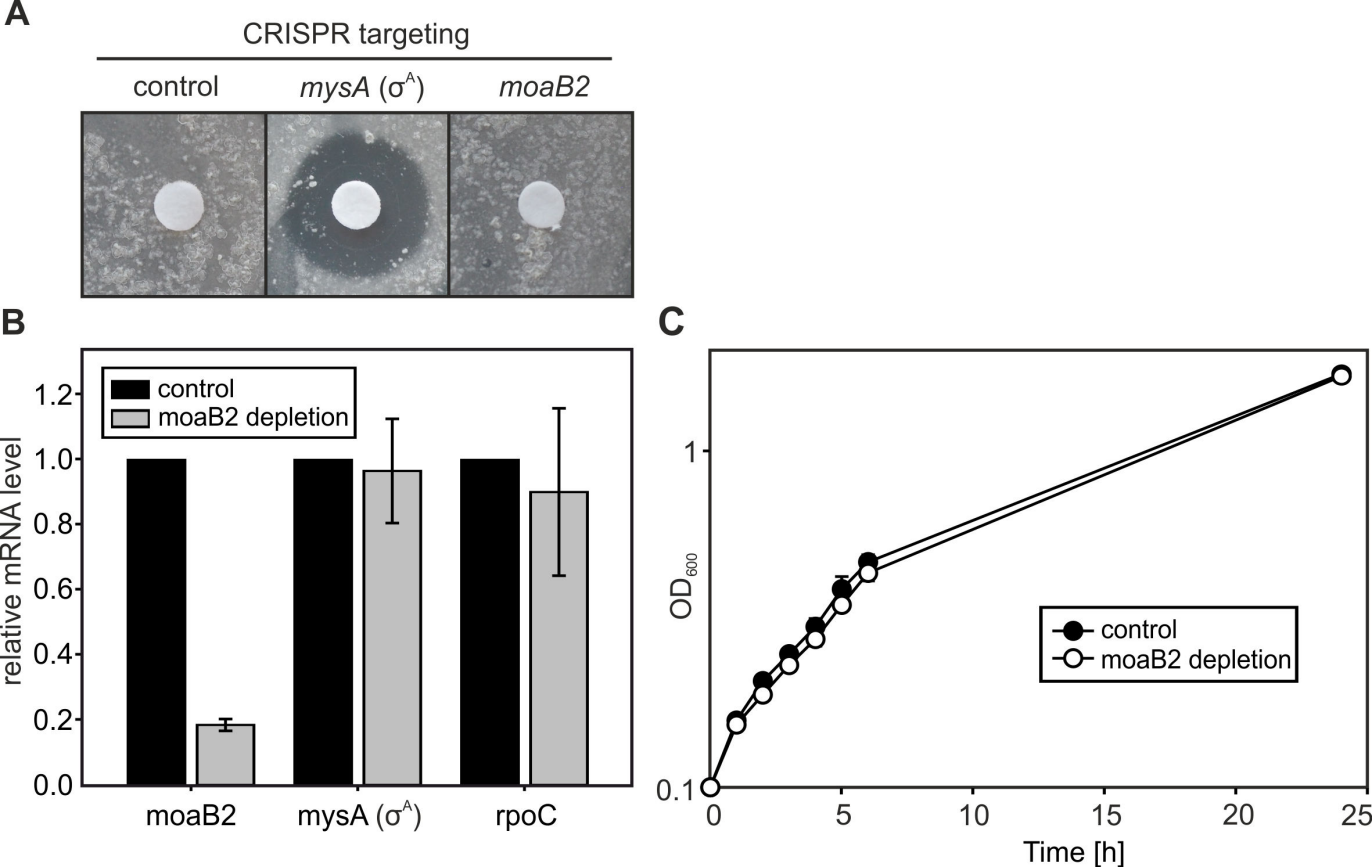
*M. smegmatis* MoaB2 is not essential. (**A**) The essentiality of the *M. smegmatis* MoaB2-encoding gene was tested by a CRISPR-based depletion approach. Discs soaked with ATC were placed on agar media. ATC induced expression of sgRNA and dCas9 to deplete σ^A^ (*mysA*, *MSMEG_2758*, and LK2203) mRNA, moaB2 (*MSMEG_5485* and LK2263) mRNA, and no-target negative control (LK2261). The genes that were targeted by sgRNAs are indicated above the dishes. The experiment was performed in three biological replicates with the same result. (**B**) RT-qPCR relative quantitation of three mRNAs (moaB2, mysA and rpoC) in the moaB2 CRISPR depletion strain (LK2263; gray bars) used in (**A**) compared to the control strain (LK2261; black bars, set as 1). The mRNA levels were also normalized to an external spike (RNA control introduced during the RNA extraction protocol). *mysA*: codes for σ^A^; *rpoC*: codes for the β′ subunit of RNAP. The graph shows averages from three independent experiments, and error bars show ±SD. (**C**) Growth curves in the 7H9 rich medium of the control strain (control oligo, LK2261) and the strain with depleted moaB2 (LK2263). The graph shows averages from three independent experiments, and the error bars indicate ±SD. CRISPR-based depletion of MoaB2 was induced with ATC (100 ng/mL), which was added at the beginning of growth.

To start addressing the biological role of MoaB2, we tested its essentiality *in vivo*, using a CRISPR-Cas9-based system. We knocked down the expression of either *moaB2* or *mysA* (σ^A^) (55). σ^A^ is essential for *M. smegmatis* (56) and was used as a positive control. As a negative control, we used a non-targeting genomic sequence single guide RNA (sgRNA). The promoters of sgRNA and dCas9 were ATC-inducible. We positioned ATC-soaked discs onto agar dishes where we had streaked out the respective *M. smegmatis* strains. While σ^A^ depletion resulted in large growth inhibition zones, both MoaB2-depleted and negative control strains displayed no inhibition zones (Fig. 7A). Reverse transcription-quantitative PCR (RT-qPCR) experiments confirmed that, compared to the negative control strain, both the MoaB2 and σ^A^ mRNA had been depleted (Fig. 7B; Fig. S9). Subsequent growth experiments then did not reveal any difference between MoaB2 depleted and control strains (Fig. 7C). We concluded that MoaB2 was not essential, and its depletion did not affect growth of the strain.

### MoaB2 modulates transcription *in vitro*

**Fig 8:**
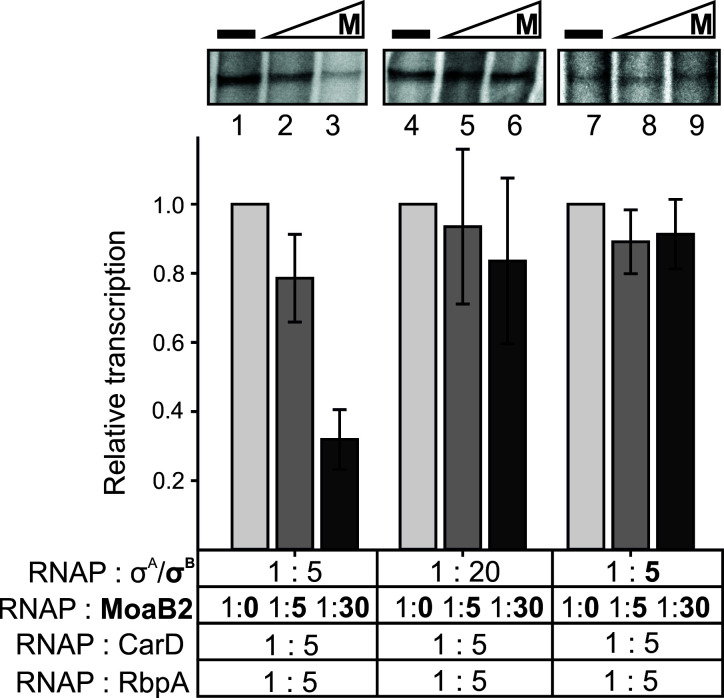
*M. smegmatis* MoaB2 modulates σ^A^-dependent but not σ^B^-dependent transcription *in vitro*. Multiple-round transcriptions were performed with RNAP (LK1853) reconstituted with σ^A^ (LK2832) at the 1:5 and 1:20 ratios or σ^B^ (LK1248) at the 1:5 ratio in the absence or presence of increasing amounts of MoaB2 (LK2936) and at the presence of CarD (LK3209) and RbpA (LK3210; indicated below the graph). Representative primary data (full gels are shown in Fig. S13B) with lane numbers are shown above the graphs (M, MoaB2). All samples were run on 7% polyacrylamide gel (for more details see Materials and Methods). The stochiometric amounts of MoaB2 refer to monomers. As promoter, the *M. smegmatis* rRNA promoter P*rrnAPCL1* was used in all panels (LK1548; full sequence is shown in Fig. S13C). All graphs show averages from three independent experiments, and the error bars indicate ±SD. Representative gel with transcription from the vector containing P*rrnAPCL1* and the “empty” vector (LK2385) is shown in Fig. S13A, demonstrating the identity of the transcript.

Next, as MoaB2 forms a complex with σ^A^, we decided to test whether MoaB2 is able to affect transcription. We performed *in vitro* multiple-round transcriptions with purified RNAP, σ^A^ or σ^B^ and MoaB2, initiating from the rRNA P*rrnAPCL1* promoter. Transcription factors RbpA and CarD, which are important also for both σ^A^- and σ^B^-dependent transcription, were included (28). Figure 8 then shows that increasing amounts of MoaB2 displayed progressively increasing inhibitory effects on σ^A^-dependent transcription, suggesting that the formation of the MoaB2-σ^A^ complex competes with the formation of the holoenzyme (lanes 1–3). Consistent with this model, an increase in the σ^A^ amount ameliorated the inhibitory effect of MoaB2 (lanes 4–6). Finally, transcriptions with RNAP associated with σ^B^ were not affected by the presence of MoaB2 (lanes 7–9), consistent with the lack of interaction between MoaB2 and σ^B^.

We concluded that MoaB2 has the potential to modulate σ^A^-dependent but not σ^B^-dependent transcription.

### MoaB2 may affect the stability of σ^A^ *in vivo*

Finally, we speculated that MoaB2 might have additional role(s) in σ^A^ regulation. We tested the hypothesis that MoaB2 affects the stability of σ^A^ in the cell. We compared MoaB2 depleted (by CRISPR Cas9) and control strains. We grew the cells to mid-exponential phase and blocked translation by the addition of streptomycin, which binds to the 30S ribosomal subunit and interferes with the complex formation between mRNA in the ribosome. We then determined the relative levels of σ^A^ and the β subunit of RNAP in both strains before and at three time points after the addition of streptomycin. Figure S10 shows that, in the control strain, the σ^A^ level did not decrease during the time course of the experiment, whereas in the MoaB2 depleted strain, the σ^A^ level decreased by the 4 h time point approximately twofold. The control protein, the β subunit of RNAP, was then about equally stable in both control and MoaB2-depleted strains. We note, however, that the MoaB2-σ^A^ interaction is more prominent in stationary phase. We attempted to determine the stability of σ^A^ also in this phase, but due to its relatively low level, we were unable to obtain reliable data. Hence, we concluded that MoaB2 might have a stabilizing effect on σ^A^, but the presented experiments are not fully conclusive.

## DISCUSSION

**Fig 9:**
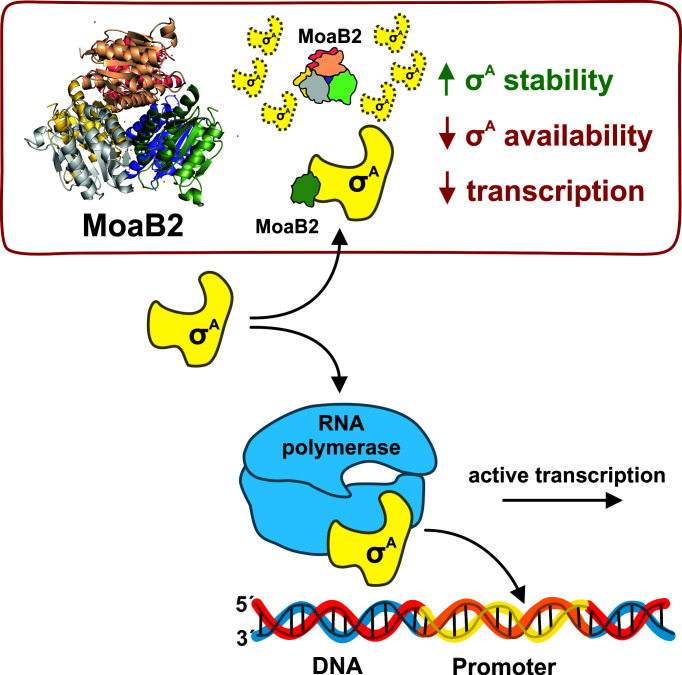
Model of interplay between MoaB2, σ^A^, and RNAP in the mycobacterial cell. A model of functional interactions between MoaB2, σ^A^, and RNAP is shown. Binding of MoaB2 to σ^A^ occurs at a ratio of 1:1 (one chain of each) and has the potential to decrease the available pool of σ^A^, likely modulating transcription by competing with the RNAP core for σ^A^. σ^A^ bound to MoaB2 is not able to bind to RNAP. MoaB2 by interacting with σ^A^ may positively affect its stability.

In this study, we have identified a new specific binding partner of *M. smegmatis* σ^A^, MoaB2, that appears not to interact with alternative σ factors. A recent study also detected MoaB2 interacting with σ^A^ from *Corynebacterium glutamicum* (57) but without further characterization. In our study, we further revealed the importance of the N-terminal domain of σ^A^ for the interaction and experimentally demonstrated the previously proposed unstructured character of this domain. We solved the 3D structure of MoaB2 by X-ray crystallography and performed functional characterization of the protein with respect to transcription, detecting its potential to modulate the process by sequestering σ^A^ and affecting its stability in the cell (Fig. 9).

### σ^A^-MoaB2 interaction

Unlike *E. coli* σ^70^_1.1_, *M. smegmatis* σ^A^_N_ has never been detected in the RNAP active site cleft. However, due to the flexible character of this domain, it cannot be excluded that σ^A^_N_ can transiently occupy this area (58). Nevertheless, it was proposed that together with the RNAP β2 domain and β′i1 (a lineage-specific insertion in β′) σ^A^_N_ may restrict the entry of DNA into the active site cleft, thereby playing a role in the formation of the open complex, an important kinetic intermediate during transcription initiation (19).

Our data show that σ^A^_N_ is involved in binding of MoaB2 to σ^A^. Surprisingly, the 60 N-terminal, predominantly positively charged aa residues of σ^A^ (Fig. 5A) do not seem to play a role in the interaction, despite the predominantly negative surface electrostatic potential of MoaB2 (Fig. S7). Rather, the _112_AATPAVATAKAA_123_ stretch, which is in the middle of an otherwise strongly acidic region between Pro61 and Pro130, seems to mediate the interaction (Fig. 6). It is apparent, however, that the interacting residues within this patch remain disordered. Possible interactions of other σ^A^ domains were not characterized at the levels of individual residues due to the size limit of the NMR experiment. Nevertheless, broadening of side-chain peaks of the σ^A^ well-ordered domains confirmed that these domains are involved in the complex with MoaB2.

Our analytical ultracentrifugation (AUC) experiments then revealed a relative 1:1 stoichiometry for the interaction between σ^A^ and MoaB2. At the excess of σ^A^, each MoaB2 protomer bound to one σ^A^ molecule. The estimated relatively high (submicromolar) binding affinity suggests a strong interaction *in vitro*. At excess of MoaB2, the sedimentation coefficient decreased, implying incomplete saturation of MoaB2 by σ^A^. However, such conditions are not expected *in vivo* due to the assumed excess of σ^A^ in the bacterial cell.

### Involvement of MoaB proteins in Moco biosynthesis

*M. smegmatis* MoaB2 has two homologs in *E. coli*, MogA and MoaB. MogA in *E. coli* serves as an adenylyltransferase that catalyzes activation of MPT for molybdenum (Mo) insertion to form Moco (33). However, it was demonstrated that MoaB is not required for Moco biosynthesis in *E. coli* despite being encoded within the largest operon, *moaABCDE*, involved in this process (31, 32). This absence of enzymatic activity is due to the presence of a glutamate instead of a catalytically important aspartate residue (Asp56 in *Pyrococcus furiosus* MoaB). This residue plays a key role in the coordination of Mg^2+^ ions during ATP-dependent MPT adenylation (50, 59). The biological role of *E. coli* MoaB thus remains enigmatic (60). Likewise, the role of *M. smegmatis* MoaB2 in Moco biosynthesis is unlikely, especially when *M. smegmatis* MoaB2 contains serine at the respective position (Ser51; Fig. S11).

MoaB and MogA homologs are not equally distributed among prokaryotes. In bacteria, species with only MogA or only MoaB or both are found. In contrast, a search in the non-redundant protein sequence database in Archaea revealed that only a limited number of organisms contain MogA, while MoaB is found in a larger number of organisms (46) (Fig. S11). This suggests that MoaB may have a yet unknown function compared to MogA in archaeal organisms. To the contrary, in Eukaryotes, it appears that most species contain MogA but not MoaB homologs.

### Genomic context of *moaB2*

In *M. smegmatis*, *moaB2* is the last gene transcribed in the *mprA-mprB-pepD-moaB2* operon. The *mprA-mprB-pepD-moaB2* locus is present and highly conserved in all mycobacterial species including *M. tuberculosis* (33). MprA and MprB are proteins of the two-component signal transduction system MprAB, where MprB is an integral-membrane sensor kinase, and MprA is a cytoplasmically localized response regulator. Interestingly, these two proteins were reported to play roles during the initial adaptive response to sub-lethal rifampicin concentration in *M. tuberculosis* (61). Rifampicin is an antibiotic that binds to the DNA/RNA channel of RNAP and inhibits transcription in its early stages (62–64). *pepD*, the third gene in the operon, encodes an HtrA-like serine protease, PepD, and is directly regulated by MprAB. PepD was shown to play roles in stress response and virulence in *M. tuberculosis* (65, 66).

### Biological role(s) of MoaB2

The genomic context of the *moaB2* gene taken together with the identified MoaB2-σ^A^ interaction, modulatory effects of MoaB2 on transcription, and its potential effects on σ^A^ stability is indicative of its role in gene expression and stress adaptation. In stationary phase, the ratio of MoaB2 to σ^A^ appeared to be higher (Fig. 5B and C). As the cellular level of *M. smegmatis* σ^A^ is lower in stationary phase than in exponential phase (67), we speculate that the sequestration of σ^A^ by MoaB2 may have more pronounced effects in stationary phase where it diminishes the already critically low level of this σ factor. The sequestration may decrease σ^A^-dependent transcription and favor the association of alternative σ factors with RNAP. At the same time, this sequestration might increase the stability of σ^A^. The effect of MoaB2 on stability of σ^A^ may then be involved in stress situations when translation is reduced and MoaB2 protects σ^A^ against degradation (Fig. 9). When conditions improve, the stored σ^A^ is available for transcription. In addition, indirect evidence suggesting a MoaB2 stabilizing effect on σ^A^ from *M. tuberculosis* was published recently (21): after artificial depletion of σ^A^ but not σ^B^, the level of the *moaB2* transcript increased, hinting at a feedback mechanism where the cell tries to counteract the loss of σ^A^ by increasing expression of a factor that enhances its stability.

By binding to the primary σ factor, MoaB2 may be analogous to Rsd of *E. coli* that binds to σ^70^ (68, 69). Rsd, structurally unrelated to MoaB2, affects the availability of σ^70^ and competition of σ factors for RNAP, especially in stationary phase (70). Although the deletion of Rsd has only minor effects on gene expression, it was shown that its function is complemented by 6S RNA, a small RNA (sRNA) that binds to and sequesters the RNAP-σ^70^ holoenzyme. Rsd also modulates expression of 6S RNA, and this crosstalk facilitates the activity of σ^38^ (71), a stress σ factor in *E. coli* (72).

In mycobacteria, no 6S RNA but Ms1 sRNA is found. Ms1, unlike 6S RNA, binds to the RNAP core and not the primary σ factor-containing holoenzyme (67, 73). Deletion of the Ms1-encoding gene affects the levels of RNAP (74). It will be of interest to determine the genome-wide effects of MoaB2 (sequestration of σ^A^) on gene expression under normal and stress conditions and also explore these effects in combination with Ms1 (sequestration of the RNAP core) and address a potential interplay between MoaB2 and the other proteins encoded within the *mprA-mprB-pepD-moaB2* operon.

To conclude, this study has identified a new binding partner of the mycobacterial primary σ factor, σ^A^, and established a basis for further investigation of its interaction with the transcription machinery and effects on gene expression. We speculate that compounds strengthening the MoaB2-σ^A^ interaction may be developed to compromise gene expression of the bacterium.

## MATERIALS AND METHODS

### Bacterial strains, plasmids, and oligonucleotides

For a detailed description of individual strains, plasmids, and oligonucleotides, see List of strains and plasmids (Table S5) and List of oligonucleotides (Table S6) in Supplementary Data.

### Construction of *M. smegmatis* strains: FLAG-tagging at the native locus

#### σ^A^-FLAG

The NEBuilder Assembly Tool (NEB New England Biolabs; https://nebuilder.neb.com/) was used to design primers to create 1 × FLAG tag σ^A^ (*MSMEG_2758=mysA*) at its endogenous locus (1× FLAG-tag was fused to the C-terminus of the protein). Final construct consisted of a hygromycin resistance cassette (HYG; LK1463) flanked by left and right “arms”: left arm (LA)—approx. 500 bp long region homologous to the 3′ terminal part of the *mysA* gene and containing the sequence encoding the affinity tag (1× FLAG-tag: DYKDDDDK); right arm (RA)—approx. 500 bp long region homologous to the sequence following the 3′ end of the targeted gene. DNA fragments (LA, HYG, and RA) were amplified by PCR with Q5 High-Fidelity DNA Polymerase (NEB) using primers #3295 + #3296, #3297 + #3306, and #3307+#3,308 (σ^A^-FLAG). For LA amplification, *M. smegmatis* mc^2^ 155 chromosomal DNA (purified from LK3016 with ChargeSwitch gDNA Mini Bacteria Kit, Invitrogen) was used as the template. For HYG amplification, plasmid containing HYG (LK1463) served as the template. For RA, the respective DNA fragment was synthesized by GeneArt Service (Invitrogen GeneArt services, ThermoFisher Scientific) and this DNA was used for PCR amplification. Fragments were then assembled into the pUC18 plasmid cloning vector using Gibson Assembly Cloning Kit (NEB). The mixture was transformed into NEB 5-alpha competent *E. coli* cells (NEB) with the standard heat-shock transformation protocol. Transformants were grown on Luria-Bertani (LB) agar supplemented with ampicillin (100 µg/mL). This yielded construct pUC18-σ^A^-FLAG (LK3182). The sequence of the construct was verified by sequencing. Construct was then cleaved with restriction enzymes BamHI/HindIII (NEB) from plasmid and transformed by electroporation into *M. smegmatis* mc^2^ 155 electrocompetent cells, prepared and performed as described previously (75, 76), containing the pJV53/*kan* integrating plasmid vector (LK1321), and plated on hygromycin (50 µg/mL) 7H10 plates (77, 78). Then, the σ^A^-FLAG strain was cured from pJV53 by passaging without kanamycin, resulting in the final *M. smegmatis* strain LK3207 (σ^A^-FLAG) with 1× FLAG-tag incorporated at the native locus.

### Construction of *M. smegmatis* strains: FLAG-tagging at the ectopic locus

σ^A^_Δ60aaN_-FLAG, σ^A^_ΔN_-FLAG, σ^B^-FLAG, σ^E^-FLAG, σ^F^-FLAG, σ^G^-FLAG, and σ^H^-FLAG.

The genes coding for the σ^A^_Δ60aaN_-1×FLAG (*MSMEG_2758* with deletion of 60 amino acids from the N terminus), σ^A^_ΔN_-3×FLAG (*MSMEG_2758*_Δ_*_N_*), σ^B^-3×FLAG (*MSMEG_2752),* σ^E^-3×FLAG (*MSMEG_5027*), σ^F^-3×FLAG (*MSMEG_1804*), σ^G^-3×FLAG (*MSMEG_0219*), and σ^H^-3×FLAG (*MSMEG_1914*) proteins were amplified by PCR using Phusion DNA Polymerase (NEB) with primers #5033 + #5034 (σ^A^_Δ60aaN_), #2901 + #2340 (σ^A^_ΔN_), #2337 + #2392 (σ^B^), #2343 + #2395 (σ^E^), #2345 + #2396 (σ^F^), #2347 + #2397 (σ^G^), and #2349 + #2398 (σ^H^) and *M. smegmatis* mc^2^ 155 chromosomal DNA (LK3016) as the template. The 3×FLAG-tag encoding sequence was added to respective genes in two steps. First, the gene was amplified in the first PCR with the reverse primer carrying part of 3×FLAG sequence, and the rest of 3×FLAG sequence was then added in the second PCR. The two fragments were combined and fused by PCR with specific forward primers and the #2385 reverse primer. In the case of cloning σ^A^_Δ60aaN_, 1×FLAG-tag was encoded in the reverse primer. Subsequently, the genes were ligated into the pTetInt integrative plasmid (79) via NdeI/HindIII or NdeI/PacI restriction sites. The constructs were verified by sequencing. The resulting plasmids were transformed into *M. smegmatis* mc^2^ 155 (LK3016) cells by electroporation resulting in strains LK4207 (σ^A^_Δ60aaN_-1×FLAG), LK2463 (σ^A^_ΔN_-3×FLAG), LK2077 (σ^B^-3×FLAG), LK2157 (σ^E^-3×FLAG), LK2159 (σ^F^-3×FLAG), LK2161 (σ^G^-3×FLAG), and LK2160 (σ^H^-3×FLAG).

*M. smegmatis* containing ectopic σ^A^-1×FLAG (LK2073) was prepared previously in our lab (80).

### Construction of *E. coli* strains for overexpression of σ^A^

Gene coding for the *M. smegmatis* protein σ^A^ was cloned into pET28-MBP-TEV vector (a gift from Zita Balklava & Thomas Wassmer; Addgene plasmid #69929; http://n2t.net/addgene:69929) (81) by the method of Restriction Free PCR cloning (82). Briefly, gene for protein σ^A^ (*MSMEG_2758*, *mysA*) was amplified by PCR using Q5 High-Fidelity 2× Master Mix (NEB) with primers #TK1+#TK2 (*MSMEG_2758*, *mysA*) from plasmid pET22b containing cloned σ^A^ (LK1740) (80) used as the template. The cleavage site for TEV protease was placed at the 5′ end of the gene construct. Target was amplified under these conditions: initial denaturation (98°C, 2 min), 30 cycles of denaturation (98°C, 10 s), annealing (65°C, 20 s), and amplification (72°C, 2 min) followed by final extension (72°C, 5 min). Amplified gene for σ^A^ with 5′ overlapping regions from the desired insertion sites at pET28-MBP-TEV was used as a primer for the second-cloning PCR reaction. PCR reaction was performed in a final volume of 20 µL containing Q5 High-Fidelity 2× Master Mix (NEB), 20 ng pET28-MBP-TEV plasmid DNA, and 100 ng of purified amplified gene for σ^A^. PCR-cloning reaction was done under these conditions: single denaturation step (98°C, 5 min), followed by 35 cycles of denaturation (98°C, 1 min), annealing (55°C, 45 s), elongation (72°C, 8 min), and final elongation step lasted for 10 min at 72°C. After the parental plasmid elimination by DpnI treatment (1 µL of the enzyme to the PCR reaction mixture), 4 µL of the PCR product was transformed to the *E. coli* DH5α cells. The resulting protein fusion of MBP-σ^A^ thus has 6× His tag at the N-terminus. MBP is cleavable by TEV protease [MBP construct end with sequence MHHHHHHVNSLEENLYFQG followed by the second amino acid of gene *MSMEG_2758* (*mysA*)]. The resulting construct (LK2844) was verified by sequencing and then transformed into *E. coli* Lemo21 (DE3) cells (NEB) resulting in expression strains LK2832 (σ^A^).

### Construction of *E. coli* strains for overexpression of MoaB2 and CarD

Gene constructs coding for the N-terminally His-tagged *M. smegmatis* proteins MoaB2 and CarD were amplified by PCR using Q5 High-Fidelity DNA Polymerase (NEB) with primers #3632 + #3633 (*MSMEG_5485*, *moaB2*) and #3775 + #3776 (*MSMEG_6077*, *carD*) from *M. smegmatis* mc^2^ 155 chromosomal DNA (LK3016) as the template. The cleavage site for TEV protease was placed at the 5′ end of the gene construct. Amplified genes for MoaB2 and CarD were cloned into the Champion pET302/NT-His expression vector using the PCR by the method of Restriction Free PCR cloning (82). PCR was the same as described in Construction of *E. coli* strains for overexpression of σ^A^. Both PCR products were separately transformed to the *E. coli* DH5α cells. The resulting proteins thus have 6× His-tags at their N-termini, cleavable with TEV protease [protein construct starts with sequence MHHHHHHVNSLEENLYFQG followed by the first amino acid of the gene *MSMEG_5485* (*moaB2*) or *MSMEG_6077* (*carD*)]. The resulting constructs (LK2938–MoaB2; LK3679–CarD) were verified by sequencing. Plasmids were then transformed into *E. coli* Lemo21 (DE3) cells (LK2678) resulting in expression strains LK2936 (MoaB2) and LK3209 (CarD).

*Msm* RNAP-His (LK1853) and RbpA (LK3210) were prepared previously in our lab (58).

### Construction of *E. coli* strain for overexpression of MoaB2 for crystallography and σ^B^

Gene construct coding for the C-terminally His-tagged *M. smegmatis* proteins MoaB2 and σ^B^ were amplified by PCR using Q5 High-Fidelity DNA Polymerase (NEB) with primers #3189 + #2472 (*MSMEG_5485*, *moaB2*) and #1153+#1154 (*MSMEG_2752*, σ^B^) from *M. smegmatis* mc^2^ 155 chromosomal DNA (LK3016) as the template. Amplified genes for MoaB2 and σ^B^ were cloned into the pET22b expression vector and transformed into *E. coli* DH5α cells. The resulting constructs were verified by sequencing. Plasmids were then transformed into *E. coli* BL21 (DE3) cells (LK625) resulting in expression strains LK2615 (MoaB2) and LK1248 (σ^B^).

### Media and growth conditions for *M. smegmatis* strains

*M. smegmatis* strains with “no FLAG” (wt, LK3016) and strains containing FLAG-tags at the native loci in the genome [σ^A^-FLAG (LK3207), RNAP-FLAG (LK2740)] were grown in Middlebrook 7H9 medium with 0.2% glycerol and 0.05% Tween 80 at 37°C. Cells were harvested in exponential (OD_600_ ∼ 0.5; 6 h of cultivation) or early stationary (OD_600_ ∼ 2.5–3.0, 24 h of cultivation) phase of growth. When required, media were supplemented with hygromycin (50 µg/mL) or streptomycin (100 µg/mL) or kanamycin (20 µg/mL). Expression of proteins with the FLAG-tag at the ectopic locus [σ^A^-FLAG (LK2073), σ^A^_ΔN_-FLAG (LK2463), RNAP-FLAG (LK1468), σ^B^-FLAG (LK2077), σ^E^-FLAG (LK2157), σ^F^-FLAG (LK2159), σ^G^-FLAG (LK2161), and σ^H^-FLAG (LK2160)] was induced by ATC (10 ng/mL) added after 3 h of growth. Cells were harvested in exponential phase as described above. In stationary phase, ATC (10 ng/mL) for induction was added at OD_600_ ∼ 1.5, and cells were harvested in early stationary phase as described above.

### Media and growth conditions for *E. coli* strains

*E. coli* strains were grown in LB media at 37°C, supplemented, when needed, with ampicillin (100 µg/mL), chloramphenicol (30 µg/mL), or kanamycin (50 µg/mL). Isopropyl β-D-thiogalactoside (IPTG, Amresco) was added to induce expression of proteins where indicated.

In the case of ^15^N-labeling for the NMR study of σ^A^_N_, the *E. coli* strains were grown in M9 minimal medium containing ^15^NH_4_Cl as sole source of nitrogen using standard protocol at 37°C.

Competent *E. coli* strain DH5α (LK13) used for cloning, or *E. coli* Lemo21 (DE3) cells (LK2678) and *E. coli* BL21 (DE3) cells (LK625), used for overexpression of proteins, were prepared according to the method of (83).

### Protein purification for biochemical assays

The strains of *E. coli* Lemo21 (DE3) cells containing expression vectors for expression of MoaB2 (*MSMEG_5485*; LK2936), CarD (*MSMEG_6077*; LK3209), σ^B^ (*MSMEG_2752*; LK1248), and RbpA (*MSMEG_3858*; LK3210) were grown in LB medium at 37°C until OD_600_ = 0.5. Expression of MoaB2 and CarD was then induced with 800 µM IPTG and expression of σ^B^ and RbpA was then induced with 500 μM IPTG. Cultures after induction were grown for 4 h at room temperature, harvested by centrifugation, and pelleted. Expression vector pET28-MBP-TEV with σ^A^ (*MSMEG_2758*; LK2832) was grown in LB medium at 37°C until the OD_600_ = 0.6. The growth temperature was then reduced to 20°C. After 30 min, expression of σ^A^ was induced by the addition of 500 µM IPTG, and the culture was grown overnight at 20°C. In the morning, cells expressing σ^A^ were harvested by centrifugation and pelleted. Pellets of cells containing MoaB2, σ^A^, σ^B^, and RbpA were washed, resuspended in *P* buffer containing 300 mM NaCl, 50 mM Na_2_HPO_4_, 5% glycerol, and 3 mM β-mercaptoethanol. In the case of CarD, T buffer (300 mM NaCl, 50 mM Tris-HCl, pH 7.5, 5% glycerol, and 3 mM β-mercaptoethanol) was used during whole isolation. Pellets were lysed using sonication [Sonopuls HD3100, Bandelin (Germany); 50% amplitude, 15 × 10 s pulse, and 1 min pause]. Cell debris was removed by centrifugation, and supernatant was mixed with 1 mL Ni-NTA Agarose (Qiagen) and incubated for 90 min at 4°C with gentle shaking. Ni-NTA Agarose with bound MoaB2, CarD, σ^A^, σ^B^ or RbpA was loaded on a Poly-Prep Chromatography Columns (Bio-Rad), washed with *P* buffer/T buffer and then, with *P* buffer/T buffer supplemented with 30 mM imidazole, and eluted with *P* buffer/T buffer containing 400 mM imidazole. Fractions containing individual proteins were pooled, based on an SDS-PAGE stained with SimplyBlue SafeStain (Invitrogen) analysis, and dialyzed for 20 h into the cleavage buffer [50 mM Tris-HCl, pH 8.0, 100 mM NaCl, 0.5 mM EDTA, 0.5 mM dithiothreitol (DTT), and 5% glycerol], except for σ^B^. σ^B^ was direclty dialyzed into the storage buffer (50 mM Tris-HCl, pH 8.0, 100 mM NaCl, 50 % glycerol, 3 mM β-mercaptoethanol) and stored at –20 °C. TEV protease was then added to dialyzed proteins (MoaB2, CarD, σ^A^, and RbpA) at a TEV protease:protein ratio 1:20. The cleavage was allowed to proceed for 8–12 h at 4°C. Cleaved NT-His tags and TEV protease (His-tagged) were removed from protein solutions with Ni-NTA Agarose. One milliliter of Ni-NTA Agarose was added to MoaB2, CarD, σ^A^ or RbpA and incubated for 90 min at 4°C with gentle shaking. Mixtures of Ni-NTA Agarose with bound NT-His tag and cleaved MoaB2, CarD, σ^A^ or RbpA were loaded on Poly-Prep Chromatography Columns (Bio-Rad), and the flow through was captured. Flow through containing individual proteins was analyzed with SDS-PAGE stained with SimplyBlue SafeStain (Invitrogen), and individual proteins were then separately dialyzed into the starting buffer (buffer A) containing 50 mM Tris-HCl, pH 8.0, 100 mM NaCl, 1 mM EDTA, 5% glycerol, and 3 mM β-mercaptoethanol for 20 h. Proteins were further purified using HiTrap Q HP anion exchange chromatography column (Cytiva) equilibrated in starting buffer (buffer A); elution saline gradient: 0.0–0.5 M of sodium chloride in the starting buffer. Flow rate, 0.5 mL/min. Fractions of 0.5 mL were collected. Individual fractions from each protein were analyzed on SDS-PAGE gel. Fractions containing pure protein were pooled and dialyzed for 20 h into the storage buffer containing 50 mM Tris-HCl, pH 8.0, 100 mM NaCl, 50% glycerol, and 3 mM β-mercaptoethanol. Isolated proteins were stored at –20°C. Novex Sharp Pre-stained Protein Standard (Invitrogen) was used as a protein marker on each SDS-PAGE, unless stated otherwise.

*M. smegmatis* σ^A^_N_ (*MSMEG_2758*; LK2864) was expressed and purified from *E. coli* BL21 (DE3) with vector σ^A^_N_-MBP-His, Lemo21 (DE3) as described for σ^A^ (*MSMEG_2758*; LK2832).

*M. smegmatis* RNAP was purified from *E. coli* BL21 (DE3) containing plasmid pRMS4 (LK1853) as described previously (58).

### CRISPR Cas9 knockdown strains preparation

SgRNA oligonucleotides #2484 + #2485 targeting *MSMEG_5485* (*moaB2*) and sgRNA oligonucleotides #2455 + #2456 targeting *MSMEG_2758* (*mysA*, σ^A^) were designed and cloned into pLJR962 according to the published protocol (55). Sequence-verified constructs were electroporated into *M. smegmatis* mc^2^ 155 (LK3016) resulting in strains LK2263 (*MSMEG_5485*, *moaB2* knockdown) and LK2203 (*MSMEG_2758*, σ^A^ knockdown). Strain containing CRISPR Cas9 negative control, non-targeting control sgRNA (LK2261), was used previously in our lab (74).

### Growth conditions of CRISPR Cas9 knockdown strains

CRISPR Cas9 knockdown strains [negative non-targeting control sgRNA (LK2261), *moaB2* knockdown (LK2263), and σ^A^ knockdown (LK2203)] for determination of essentiality of the genes were grown to mid-exponential phase and plated onto 7H10 solid media with or without discs soaked with ATC (100 ng/mL). Plates were incubated at 37°C for 3–4 days, visually monitored.

CRISPR Cas9 knockdown strains [negative control non-targeting control sgRNA (LK2261), *moaB2* knockdown (LK2263)] were grown at 37°C in Middlebrook 7H9 medium with 0.2% glycerol, 0.05% Tween 80, and ATC (100 ng/mL). For growth curves and for RNA isolation from stationary phase, cells were grown for 24 h; OD_600_ was measured each hour between 0–6 h and after 24 h. An aliquot of 25 mL of cells from stationary phase was then cooled on ice, pelleted, and immediately frozen at –80°C. For western blot analysis, cells were grown until mid-exponential phase (OD_600_ ∼ 0.6; 8 h of cultivation) when streptomycin (100 µg/mL) was added to stop translation. Aliquots of 20 mL were harvested at time 0 (just before the addition of streptomycin) and 4 h after the addition of the streptomycin, pelleted, and immediately frozen at –80°C.

### RNA isolation

Prior to RNA extraction from strains LK2263 (*moaB2* knockdown) and LK2203 (σ^A^ knockdown), an RNA Spike [recovery marker: Plat mRNA (718 nt) from *M. musculus* prepared from pJET_Plat_IVTs; the sequence of the fragment is in Fig. S12] was added, and the amount of which was calculated based on cell density (1.86 ng was added to 30 mL of culture at OD_600_ = 0.5). The RNA spike was a kind gift from Dr. Radek Malík, IMG, Prague. Total RNA was then extracted by resuspending the pellets in 240 µL TE (pH 8.0) plus 60 µL lysis buffer (50 mM Tris-HCl, pH 8.0, 500 mM LiCl, 50 mM EDTA, pH 8.0, and 5% SDS) and 600 µL of acidic phenol (pH ~ 3):chloroform (1:1). Lysates were sonicated for 1 min on ice in fume hood and centrifuged. The aqueous phase was extracted two more times with acidic phenol (pH ~ 3):chloroform (1:1) and precipitated with ethanol. RNA was dissolved in water, treated with DNase (TURBO DNA-free Kit, Ambion) and reverse transcribed into cDNA (SuperScriptIII, Invitrogen) using random hexamers.

### Reverse transcription-quantitative PCR

RT-qPCR was used to amplify cDNA in duplicate reactions containing LightCycler 480 SYBR Green I Master and 0.5 µM primers (each). Primers #2476 + #2477 for *moaB2* (*MSMEG_5485*) were designed with the Primer3 software. Primers for σ^A^ (*MSMEG_2758*, *mysA*) #987 + #988 and *rpoC* (*MSMEG_1368*) #989 + #990 were prepared and used previously (74). Primers #3542 + #3543 were used for the RNA spike. Negative controls (no template reactions and reactions with RNA without reverse transcription) were run in each experiment. For RT-qPCR, a LightCycler 480 System (Roche Applied Science) was used. Quality of qPCR products was determined by dissociation curve analysis and the efficiency of the primers determined by standard curves. The mRNA level was quantified on the basis of the threshold cycle (Ct) for each PCR product that was normalized to the spike recovery marker value according to the formula 2^[Ct (spike)-Ct (mRNA)]^.

### Immunoprecipitation

Cells were grown as described in Media and growth conditions for *M. smegmatis* strains. 150 mL of exponential and 50 mL of early stationary phase cells were pelleted. Pellets were resuspended in 4 mL of Lysis buffer (20 mM Tris-HCl, pH 8.0, 150 mM KCl, and 1 mM MgCl_2_) containing 1 mM DTT, 0.5 mM phenylmethylsulfonyl fluoride (PMSF), and protease inhibitor cocktail P8849 (Sigma-Aldrich, 5 µL/mL) and sonicated 15 × 10 s with 1 min pauses on ice and centrifuged. One milliliter of stationary and 1.5 mL of exponential phase cells lysates were incubated overnight at 4°C with 25 µL of anti-FLAG M2 Affinity Agarose Gel (Sigma, A2220). Agarose gel beads with the captured protein complexes were washed 4× with 0.5 mL of lysis buffer. FLAG-tagged proteins were eluted with 60 µL of 3 × FLAG Peptide [Sigma F4799; diluted in Tris-buffered saline (TBS) to a final concentration of 150 ng/mL]. Ten microliters of each elution was resolved on SDS-PAGE gel and stained with SimplyBlue SafeStain (Invitrogen).

For immunoprecipitation of σ^A^ using antibody against σ^70^ (anti-σ^70^, clone name 2G10, BioLegend, cat. No. 663208), *M. smegmatis* stationary phase cells (wt, LK3016) were pelleted and resuspended in 20 mM Tris-HCl pH 8.0, 150 mM KCl, 1 mM MgCl_2_, 1 mM DTT, 0.5 mM PMSF and protease inhibitor cocktail P8849 (Sigma-Aldrich, 5 µg/mL) and sonicated 15 × 10 s with 1 min pauses on ice and then centrifuged. Subsequently, 300 µg (protein) of cell lysates were incubated for 2 h at 4°C with 20 µL of Dynabeads Protein A (Invitrogen) coated either with 4 µg mouse monoclonal antibody to σ^70^ or 10 µg mouse nonspecific IgG (Sigma-Aldrich) used as a negative control. The captured complexes were washed 4× with 20 mM Tris-HCl pH 8, 150 mM KCl, and 1 mM MgCl_2_. The beads were incubated in SDS sample buffer for 5 min at 95°C, and eluted proteins were resolved on SDS-PAGE gel and stained with SimplyBlue SafeStain (Invitrogen).

### LC-MS/MS analysis

After immunoprecipitation, proteins were digested with 0.1 µg of trypsin solution in 50 mM ammonium bicarbonate at 37°C for 16 h. The resulting peptides were separated on an UltiMate 3,000 RSLCnano system (Thermo Fisher Scientific) coupled to an Orbitrap Fusion Lumos mass spectrometer (Thermo Fisher Scientific). The peptides were trapped and desalted with 2% acetonitrile in 0.1% formic acid at a flow rate of 30 µL/min on an Acclaim PepMap100 column [5 µm, 5 mm by 300 µm internal diameter (ID); Thermo Fisher Scientific]. The eluted peptides were separated using an Acclaim PepMap100 analytical column (2 µm, 50 cm by 75 µm ID, ThermoFisher Scientific). The 125 min elution gradient at a constant flow rate of 300 nL/min was set to 5% of phase B (0.1% of formic acid in 99.9% of acetonitrile) and 95% of phase A (0.1% of formic acid) for 1  min, after which the content of acetonitrile was gradually increased. The Orbitrap mass range was set from m/z 350 to 2,000 in the MS mode, and the instrument in data-dependent acquisition mode acquired high-energy collisional dissociation (HCD) fragmentation spectra for ions of m/z 100–2,000.

### Protein identification and quantification

MaxQuant with Andromeda search engine (version 1.6.3.4; Max-Planck-Institute of Biochemistry, Planegg, Germany) was utilized for peptide and protein identification with databases of the *M. smegmatis* proteome (downloaded from UniProt on 20th of December 2019) and common contaminants. Perseus software (version 1.6.2.3; Max-Planck-Institute of Biochemistry) was used for the label-free quantification of three biological replicates of σ^A^-FLAG as compared to negative control (three biological replicates of wild-type strain without any tag). The same analysis was used for the comparison of σ^A^_ΔN_-FLAG as compared to negative control. The identified proteins were filtered for contaminants and reverse hits. Proteins detected in the data were filtered to be quantified in at least two of the triplicates in at least one condition (557 proteins in σ^A^-FLAG and 1,262 proteins in σ^A^_ΔN_-FLAG). The data were processed to compare the abundance of individual proteins by statistical tests in the form of student’s *t*-test and resulted in a volcano plot comparing the statistical significance (two-sided *P*-value) and protein-abundance difference (fold change). Volcano plots were created and labeled using VolcaNoseR web app (84). The mass spectrometry proteomics data have been deposited to the ProteomeXchange Consortium (85) via the PRIDE (86) partner repository with the data set identifier PXD039468 and 10.6019/PXD039468.

### Promoter DNA construction for *in vitro* transcription assay

The DNA fragment containing the σ^A^-dependent promoter P*rrnAPCL1* (−69/+120, +1G) was amplified from *M. smegmatis* mc^2^ 155 chromosomal DNA (LK3016) with primers #1474 + #1475 by PCR. The fragment was cloned into p770 [pRLG770, as described previously (87)] using EcoRI/HindIII restriction sites (DNA sequence with annotation is shown in Fig. S13C), transformed into *E. coli* DH5α competent cells, and the construct was verified by sequencing, yielding plasmid LK1548. Supercoiled plasmid containing the promoter was purified for *in vitro* transcription assays using Wizard Midiprep Purification System (Promega) and subsequently phenol-chloroform extracted, precipitated with ethanol, and dissolved in water.

### *In vitro* transcription assay

*In vitro* transcriptions were performed with σ^A^-dependent promoter P*rrnAPCL1*, an *M. smegmatis* ribosomal RNA promoter (Fig. S13C) (58). Multiple-round transcription assays were carried out essentially as described previously (88, 89) unless stated otherwise. First, reactions were carried out in 10 µL: 250 ng of supercoiled DNA template P*rrnAPCL1* (LK1548), transcription buffer (40 mM Tris-HCl, pH 8.0, 10 mM MgCl_2_, and 1 mM DTT), 0.1 mg/mL BSA, 50 mM KCl, and NTPs [200 µM ATP and CTP; 5 mM GTP; 10 µM UTP; 2 mM of radiolabeled (α^32^P)-UTP]. Transcriptions were initiated by 2 µL of reconstituted proteins [(σ^A^ ± MoaB2) + (RNAP + CarD + RbpA) for lanes 1–6 in Fig. 8 or (σ^B^ ± MoaB2) + (RNAP + CarD + RbpA) for lanes 1–6 in Fig. 8] yielding a final volume of 10 µL. The final concentrations of the reconstituted proteins in Fig. 8 and Fig. S13 were: RNAP (LK1853), 0.06 µM; σ^A^ (LK2832), 0.3 µM or 1.2 µM; MoaB2 (LK2936), 0.03 µM or 1.8 µM; σ^B^ (LK1248), 0.3 µM; CarD (LK3209), 0.3 µM; and RbpA (LK3210), 0.3 µM. Reconstitutions were carried out for 15 min at 37°C. *In vitro* transcriptions were allowed to proceed for 10 min at 37°C. Formamide stop solution (95% formamide, 20 mM EDTA, pH 8.0, 0.03% bromophenol blue, and 0.03% xylene cyanol FF) (89) was added to stop the reaction. Samples were denaturated for 5 min in 95°C and then loaded on polyacrylamide (PAA) gel (7% PAA, 0.1% APS, and 0.1% TEMED). Gel was run for 30 min at 30 W. Gel was dried for 30 min at 80°C, cooled down, and exposed overnight on BAS storage phosphor screen (Fujifilm). Subsequently, the screen was scanned using Amersham Typhoon 5 Biomolecular Imager (Cytiva) with phosphor imaging emission filter 390 BP. The signal was quantified with the QuantityOne (Bio-Rad) software.

### Western blotting

Pellets of CRISPR Cas9 knockdown strains *moaB2* (LK2263) and negative control (LK2261) were resuspended in 4 mL of Lysis buffer [20 mM Tris-HCl, pH 8.0, 150 mM KCl, 1 mM MgCl_2_, with 1 mM DTT, 0.5 mM PMSF, protease inhibitor cocktail P8849 (Sigma-Aldrich, 5 µg/mL)] and sonicated 3 × 1 min [50% amplitude, Sonopuls HD3100, Bandelin (Germany)] on ice and centrifuged. Concentrations of proteins in lysates were measured using Bradford or Qubit 4 Fluorometer (ThermoFisher Scientific). Equal amounts of proteins (30 µg/µL) were resolved by SDS-PAGE and detected by western blotting on nitrocellulose membrane (Amersham Protran Premium 0.45 NC) in western blot buffer (25 mM Tris-HCl, pH 8.0, 195 mM glycine, and 20% methanol). Membrane was blocked in 1×TBS-T (20 mM Tris-HCl, pH 7.5, 150 mM NaCl, and 0.05% Tween 20) with 5% BSA for 1 h at 23°C and cut into two parts based on Novex Sharp Pre-stained Protein Standard (Invitrogen) at 80 kDa. Membranes were separately incubated with the primary antibody solution using either mouse monoclonal antibody against the β subunit of RNAP (clone name 8RB13, BioLegend, cat. No. 663903, dilution 1:1,000) or mouse monoclonal antibody against *E. coli* σ^70^ (clone name 2G10, BioLegend, cat. No. 663208, dilution 1:100, 5 µg/mL) overnight at 4°C. This antibody recognizes also *M. smegmatis* σ^A^ (90). Subsequently, membranes were washed in 1×TBS-T and placed in the secondary antibody solution using goat-anti-mouse IgG IR800 fluorescent antibody (WesternBright MCF-IR fluorescent Western blotting kit, cat. No. K-12022–010, dilution 1:10,000) for 1 h at 23°C. Membranes were washed in 1×TBS (without Tween 20), dried for few minutes, and scanned using Amersham Typhoon 5 Biomolecular Imager (Cytiva), IR long emission filter at 800 nm excitation. The signal was quantified with the QuantityOne (Bio-Rad) software.

### Circular dichroism spectroscopy

CD spectra were recorded on Jasco J-815 spectrometer (Jasco, Hachioji, Japan) at 27°C in a 0.1 cm path-length quartz cuvette. Analyzed samples contained 7.0 µM σ^A^_N_ from *M. smegmatis* and σ^A^_1.1_ from *B. subtilis* [LK1345; expressed and purified as published earlier (18)] in a buffer composed of 20 mM sodium phosphate, 10 mM sodium fluoride, and pH 8.0. CD spectra were recorded from 185 to 260 nm in 15 accumulations after which the spectra were averaged. All experiments were measured with a scanning speed of 20 nm/min, data interval of 1 nm, 1 s response time, and 1 nm bandwidth.

### Protein crystallization

The strain of *E. coli* BL21 (DE3) containing expression vector for expression of MoaB2 (*MSMEG_5485*; LK2615) was grown and isolated the same as described in section Protein purification for biochemical assays—MoaB2, σ^A^, and CarD. After elution with P buffer containing 400 mM imidazole and pooling fractions containing purified MoaB2 based on SDS-PAGE, protein was dialyzed into the buffer containing 20 mM Tris-HCl, pH 8.0, 100 mM NaCl, and 3 mM NaN_3_ and concentrated using Amicon Ultra-4 centrifugation unit (Millipore). Before crystallization, the protein was centrifuged at 10,000 rpm for 10 min at 4°C. The crystallization conditions were tested using screening solutions purchased from Qiagen (Classics I, II, Lite and PACT Suite). Routinely, 100 nL of 5 mg/mL *M*. *smegmatis* MoaB2 protein solution were mixed with 100 nL of screening solution applying the vapor diffusion technique in 96-well Swissci triple-drop plates (sitting drop). Screens were set up using a Phoenix (Art Robbins) robot. Plates were sealed with a clear seal film and incubated at 20°C in a MinstrelHT + UV storage and imaging system (Rigaku). After 5 days, protein crystals were observed in the conditions containing 0.2 M calcium chloride, 14% (wt/vol) PEG 400, 0.1 M sodium-HEPES, and pH 7.5 (Qiagen; Classics Lite; Well Number 74). The crystals were extracted directly from the screen conditions, mounted onto nylon loops (Hampton Research), and flash frozen in liquid nitrogen with the addition of 50% glycerol cryo-protectant.

### Data collection and structure determination

Diffraction data were collected at the DESY beamline P14 (Hamburg, Germany) at a wavelength of 0.9762 Å with 0.05° oscillation and 0.009 s exposure per image, in 2,400 images and with crystal to detector distance of 287.47 mm using a Dectris EIGER2 CdTe 16M detector. Diffraction data were processed with MOSFLM (91) and SCALA of the CCP4 program suite (92). Data collection statistics are summarized in Supplementary Table (Table S2). The crystal structure of *M. smegmatis* MoaB2 was determined by molecular replacement with MOLREP (93) using the coordinates of *M. marinum* MoaB2 (PDB id: 3rfq) (44) as a search model. Structure refinement was performed with REFMAC5 (94) with 5% randomly chosen reflections that were set aside to calculate *R*_free_. The model was manually refined using COOT (95). Water molecules were added with COOT, and ligand molecules were included manually. The structure was refined at 2.53 Å resolution with final *R*_work_ and *R*_free_ of 0.192 and 0.26, respectively, and was validated using the COOT validation tools. Structural coordinates were deposited in the RCSB Protein Data Bank (PDB id: 8byr). Refinement statistics are shown in Supplementary Table (Table S2).

### Size exclusion chromatography

Proteins for SEC were prepared as described earlier in “Protein purification for biochemical assays” in Materials and Methods in this article.

All experiments were performed using 25 mM Tris-HCl, pH 7.5, 50 mM NaCl, 1 mM DTT buffer, an ÄKTA Prime instrument (Amersham Bioscience, UK), and HiLoadTM Superdex 200 Increase 10/300 Gl column (GE Healthcare) calibrated using Gel Filtration Markers Kit for Protein Molecular Weights 6,500–66,000 Da (Sigma-Aldrich, MWGF70). All SEC experiments were run at 15°C with the flow rate of 0.6 mL/min, and all chromatograms were collected at 254 nm (due to the extremely low absorbance of MoaB2 at 280 nm caused by the fact that no Trp, Tyr or Cys residues are present in it). Fractions were analyzed on SDS-PAGE. Color Prestained Protein Standard, Broad Range (New England Biolabs) was used as marker. SDS-PAGE was performed under reducing conditions using NuPAGE Bis–Tris 4%–12% gradient gels and Xcell SureLock mini–cell electrophoresis system (ThermoFisher). Electrophoresis was performed according to the manufacturer’s instructions.

σ^A^-MoaB2 complex was formed by mixing sample of σ^A^ (fractions from SEC of σ^A^ eluted at 12–13.5 mL) with the excess of MoaB2 (fractions from SEC of MoaB2 eluted at 13–14.5 mL) and left to equilibrate for 1 h at room temperature.

### Dynamic light scattering

The *M. smegmatis* MoaB2 (LK2615) protein sample at 5 mg/mL in 20 mM Tris-HCl (pH 8.0), 50 mM NaCl, and 3 mM NaN_3_ was incubated at room temperature for 30 min before the experiment and then centrifuged at 10,000 rpm for 10 min at 25°C. The dynamic light scattering experiment was performed using 10 µL of protein on a Beckman-Coulter DelsaMAX Core at a laser power of 3% and at a temperature of 25°C, with 20 scans and a measurement time of 3 s.

### MALDI mass spectrometric identification

Coomassie blue (CBB) stained protein bands were cut out from gels, chopped into small pieces, and destained using 50 mM 4-ethylmorpholine acetate (pH 8.1) in 50% acetonitrile (MeCN). After the supernatant removal, the gel pieces were washed step by step with water and MeCN and then partly dried in a SpeedVac concentrator. The proteins were digested overnight at 37°C using sequencing grade trypsin (100 ng, Promega) in a buffer containing 25 mM 4-ethylmorpholine acetate and 5% MeCN. The resulting peptides were extracted with 40% MeCN/0.2% TFA (trifluoroacetic acid).

Prior to MALDI-MS analysis, 0.5 µL of each peptide mixture was deposited on the MALDI plate, air-dried at room temperature, and overlaid with 0.5 µL of the matrix solution (α-cyano-4-hydroxycinnamic acid in 50% acetonitrile/0.1% TFA; 5 mg/mL, Sigma). Peptide spectra were measured on a 15T Solarix XR FT-ICR mass spectrometer (Bruker Daltonics) in a mass range of 500–6,000 Da and calibrated externally using a PepMix II standard (Bruker Daltonics). For protein identification, the peak lists generated using DataAnalysis 5.0 program were searched against a merged SwissProt and NCBI database subset of *M. smegmatis* proteins using in-house MASCOT v.2.6 search engine with the following settings: peptide mass tolerance of 2 ppm, maximum of missed cleavages set to one, and variable oxidation of methionine.

### NMR spectroscopy

^1^H-^15^N HSQC (53, 54), HNCACB (96) CBCA(CO)NH (97), ^15^N-edited TOCSY (98), ^15^N-edited NOESY (99), and 5DHN(CA)CONH (100) NMR spectra were recorded on 850 and 950 MHz NMR Bruker NEO HD spectrometers equipped with a 5 mm triple-resonance (^1^H-^13^C-^15^N) inverse cryogenic probe with cooled ^1^H and ^13^C preamplifiers and with *z*-axis gradients. The temperature was calibrated using a standard sample of neat methanol. Spectra were processed using NMRPipe software (101) and analyzed using SPARKY (102). The NMR sample used for partial assignment consisted of 150 µM [^13^C, ^15^N]-σ^A^_N_ (residues 1–160; LK2863), 50 mM NaPi, pH 7.0, 50 mM NaCl, 0.5 mM TCEP, 2 mM NaN_3_, and 10% D_2_O, and the spectra were recorded at 27°C. Interactions between σ^A^ and MoaB2 were studied at 20°C using 125 µM [^15^N]-σ^A^ or 125 µM [^15^N]-σ^A^ (LK2832) and 250 µM unlabeled MoaB2 (LK2936) in 50 mM HEPES, pH 7.0, 100 mM NaCl, 0.5 mM TCEP, 2 mM NaN_3_, and 6% D_2_O. The combined chemical shift changes were calculated as Δδ =[δ_H_^2^ + (0.2δ_N_)^2^]^1/2^, where δ_H_ and δ_N_ are the ^1^H and ^15^N chemical shifts, respectively.

### Analytical ultracentrifugation

AUC experiments were performed using Optima AUC analytical ultracentrifuge (Beckman Coulter) equipped with an An-50 Ti rotor. Before the experiment, σ^A^ and MoaB2 were brought to 50 mM HEPES (pH 7.5), 100 mM NaCl, 0.5 mM TCEP, and 3 mM NaN_3_ by overnight dialysis, and the buffer was used as an optical reference. σ^A^ and MoaB2 concentrations were determined using the absorbance at 280 nm (A_280_) and Bradford method and validated using amino acid analysis.

SV experiments were carried out at 20°C in standard double-sector epoxy-carbon centerpiece cells with 12 mm optical path-length (Beckman Coulter) loaded with 425 µL of both sample and reference solution. The SV experiment was done with σ^A^ (19.4 µM) and MoaB2 (77.6 µM) samples and a set of σ^A^:MoaB2 mixtures (molar ratios of 1:0.1 to 1:4, all containing 19.4 µM σ^A^). Data were collected using absorbance (280 nm) and/or interference optical systems at a rotor speed of 48,000 rpm. The data were analyzed in Sedfit 16.1 c (103) with the c(s) distribution model. For the regularization procedure, a confidence level of 0.68 was used. The partial-specific volumes of σ^A^ and MoaB2, solvent density, and viscosity were predicted using Sednterp 3 (104). GUSSI 2.1.0 was used for plotting of c(s) distributions and peak integration (105).

Additionally, the MSSV approach (106) was used to determine the relative stoichiometry of σ^A^ and MoaB2 in the complex. The spectral decomposition was performed by analyzing absorbance (280 nm) and interference data of the 1:0.3 σ^A^:MoaB2 molar ratio using the multi-wavelength discrete/continuous distribution analysis model in Sedphat 15.2b (107). Concentrations of σ^A^ and MoaB2 in the complex were determined by integrating the peak of the complex in the c_k_(s) distributions.

## Data Availability

The authors declare that all data supporting the findings of this study are available within the paper and its supplementary information files. References to data stored in specific databases (PDB, PRIDE) are provided in Materials and Methods where appropriate.

## SUPPLEMENTAL MATERIAL

Supplemental figures and tables jb.00066-24-s0001.pdf Fig. S1 to Fig. S13 Tables S2 and S4 to S6.

Table S1 jb.00066-24-s0002.xlsx Contains a list of proteins pulled down with σ^A^-FLAG (LK3207).

Table S3 jb.00066-24-s0003.xlsx Contains a list of proteins pulled down with σ^A^_ΔN_-FLAG (LK2463).

ASM does not own the copyrights to Supplemental Material that may be linked to, or accessed through, an article. The authors have granted ASM a non-exclusive, world-wide license to publish the Supplemental Material files. Please contact the corresponding author directly for reuse.
