# Supplementary material for: MoaB2, a newly identified transcription factor, binds to σA in Mycobacterium smegmatis
Source: J Bacteriol. 2024 Nov 5;206(12):e00066-24. doi: 10.1128/jb.00066-24 (PMC11656743; doi:10.1128/jb.00066-24)
Supplement: Supplemental figures and tables — Fig. S1 to Fig. S13 Tables S2 and S4 to S6. [file jb.00066-24-s0001.pdf]

Supplementary information to

MoaB2, a newly identified transcription factor, binds to σ^A in *Mycobacterium smegmatis*

Barbora Brezovská[1#], Subhash Narasimhan[2,3#], Michaela Šiková[1], Hana Šanderová[1], Tomáš Koval'[4], Nabajyoti Borah[1], Mahmoud Shoman[1], Debora Pospíšilová[1], Viola Vaňková Hausnerová[1,5], Dávid Tužinčin[2,3], Martin Černý[2,3], Jan Komárek[2,3], Martina Janoušková[1], Milada Kambová[1], Petr Halada[5], Alena Křenková[7], Martin Hubálek[7], Mária Trundová[4], Jan Dohnálek[4], Jarmila Hnilicová[1,5], Lukáš Žídek[3], Libor Krásný[1]

[1] Laboratory of Microbial Genetics and Gene Expression, Institute of Microbiology of the Czech Academy of Sciences, Prague, Czech Republic

[2] Central European Institute of Technology (CEITEC), Masaryk University, Brno, Czech Republic

[3] Faculty of Science, National Centre for Biomolecular Research, Masaryk University, Brno, Czech Republic

[4] Institute of Biotechnology of the Czech Academy of Sciences, Centre BIOCEV, Průmyslová 595, 252 50 Vestec, Czech Republic

[5] Laboratory of Regulatory RNAs, Faculty of Science, Charles University, 128 44 Prague, Czech Republic

[6] Institute of Microbiology of the Czech Academy of Sciences, Centre BIOCEV, Průmyslová 595, 252 50 Vestec, Czech Republic

[7] Institute of Organic Chemistry and Biochemistry, Czech Academy of Sciences, Prague, Czech Republic

[#] These authors should be considered joint first authors.

This file includes:

Supplementary Figures (Fig. S1 - Fig. S13)

Supplementary Tables (Table S2, S4 - S6)

Supplementary References

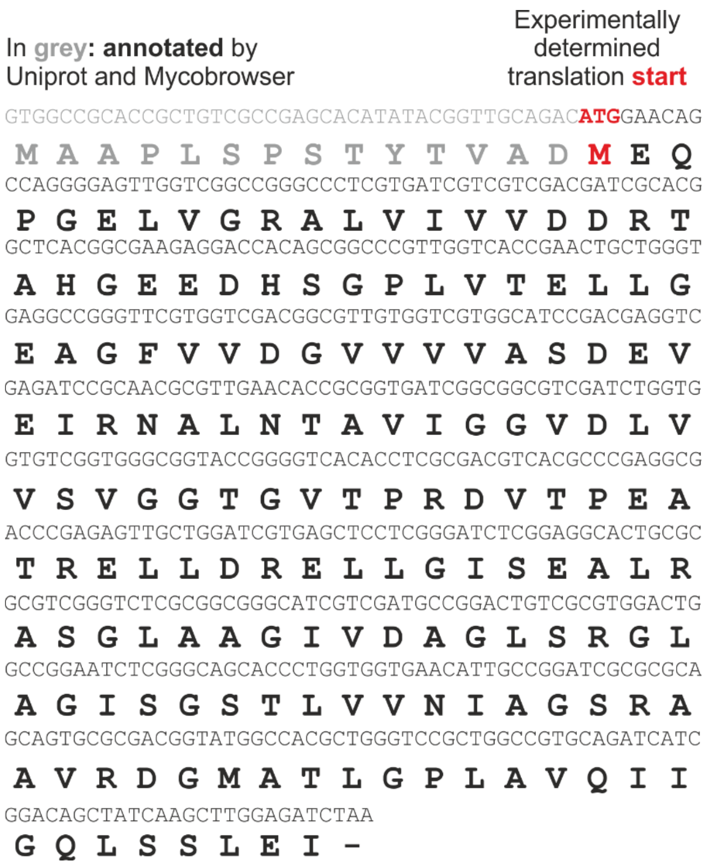

Supplementary Figure S1: MoaB2 translation start.

Nucleotide and amino acid sequences of *M. smegmatis* MoaB2 are shown. The annotated N-terminal sequence in Mycobrowser (*MSMEG_5485*) and Uniprot (A0R3I5) are shown in grey. In red color, the experimentally determined translation start site methionine (ATG) is highlighted.

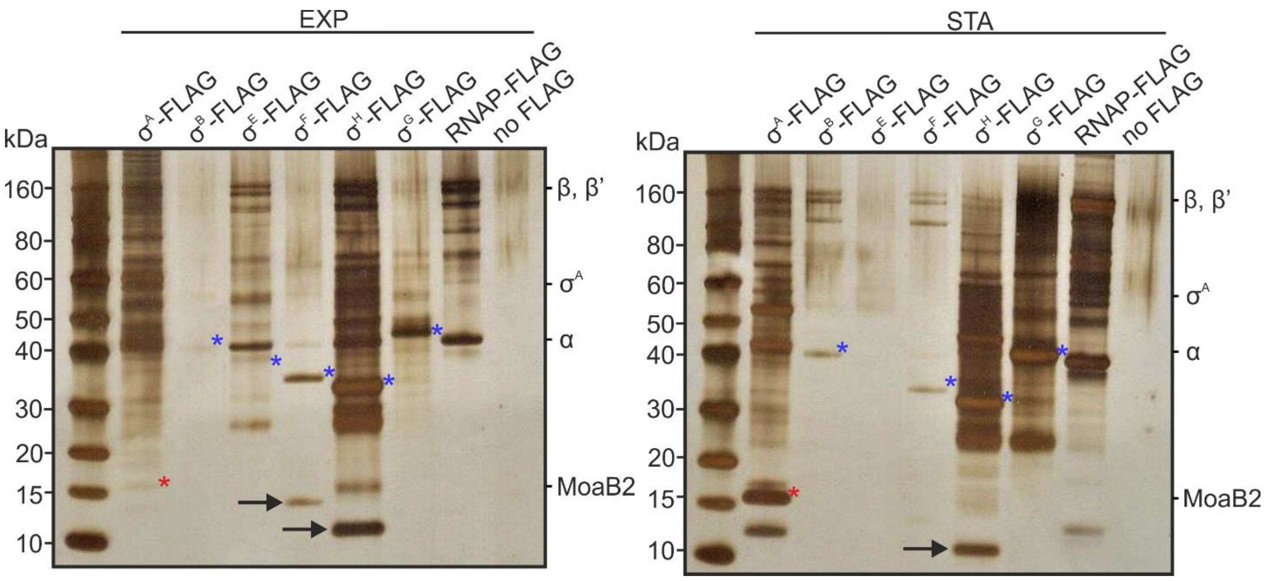

Supplementary Figure S2: *M. smegmatis* MoaB2 does not associate with alternative σ factors.

Silver stained SDS-PAGE of immunoprecipitations of FLAG-tagged sigma factors (σ^A [LK2073], σ^B [LK2077], σ^E [LK2157], σ^F [LK2159], σ^H [LK2160], σ^G [LK2161] or FLAG-tagged β´ subunit of RNAP [LK1468]) using anti-FLAG antibody. The immunoprecipitations were done from exponential (EXP) and stationary (STA) phases as indicated. The FLAG-tagged proteins were present in the genome in an additional copy under anhydrotetracycline (ATC) inducible promoter and expressed after ATC induction. The 'No FLAG' strain was used as a negative control (LK3016). Individual σ factors are marked with blue asterisks. Black arrows indicate respective anti-σ factors. MoaB2 is marked with red asterisk.

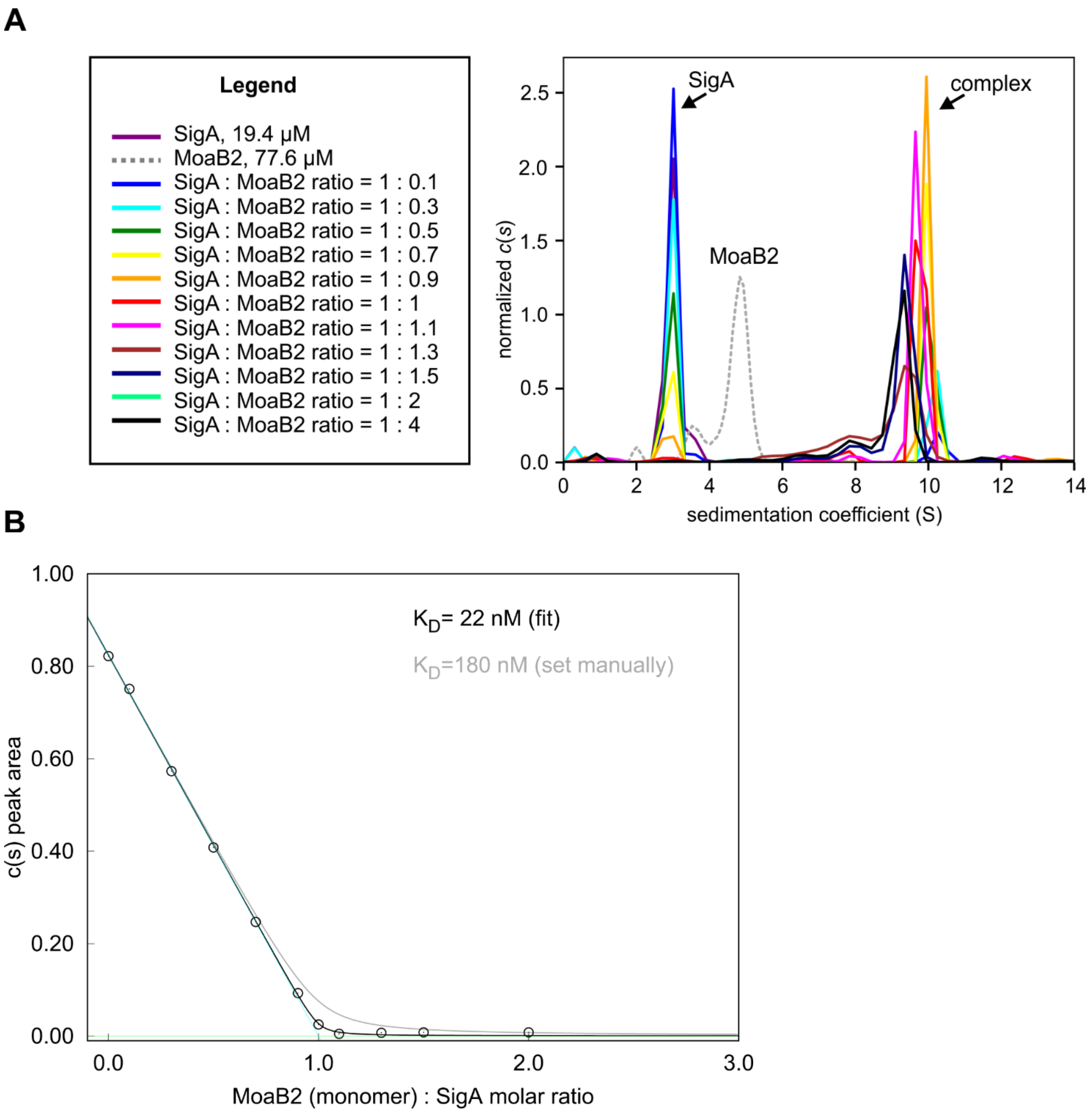

Supplementary Figure S3: Analytical ultracentrifugation (AUC) of σ^A and MoaB2.

A. Comparison of c(s) distributions determined for σ^A (19.4 µM), MoaB2 (77.6 µM) and different σ^A : MoaB2 molar ratios (all mixtures containing 19.4 µM σ^A) in 50 mM HEPES (pH 7.5), 100 mM NaCl, 0.5 mM TCEP, and 3 mM NaN_3. The sedimentation velocity experiment was performed at 20 °C and 48,000 rpm using absorbance (280 nm, solid lines) or interference (dashed line) detection.

B. Stoichiometry and affinity estimation plot: The peak area of σ^A in the c(s) distribution decreased linearly close to the stoichiometric point of 0.996±0.009, indicating a dissociation constant much lower than the used σ^A concentration. Peak area *A* proportional to the actual concentration of free σ^A ($A = a[S]$) was fitted to the equation (black curve): $A = a[S] = (a/2)\ (c_S - c_M + K_D + ((c_S - c_M + K_D)2 + 4K_D\ c_S)^{1/2})$, where c_S and c_M are total (analytical) concentrations of σ^A and MoaB2 monomer, respectively, and K_D is the dissociation constant of the complex. The result of the fit was K_D = (22 ± 7) nM and a = 0.824 ± 0.004. Note, however, that the K_D value is mostly given by a single point on the "titration curve" (close to the stoichiometric point) and we stress that the K_D value is only qualitative. In any case, K_D is much lower than the 1.8 μM concentration of MoaB2 used in the transcription experiment presented in **Fig. 8** (see gray curve, simulated for K_D = 180 nM).

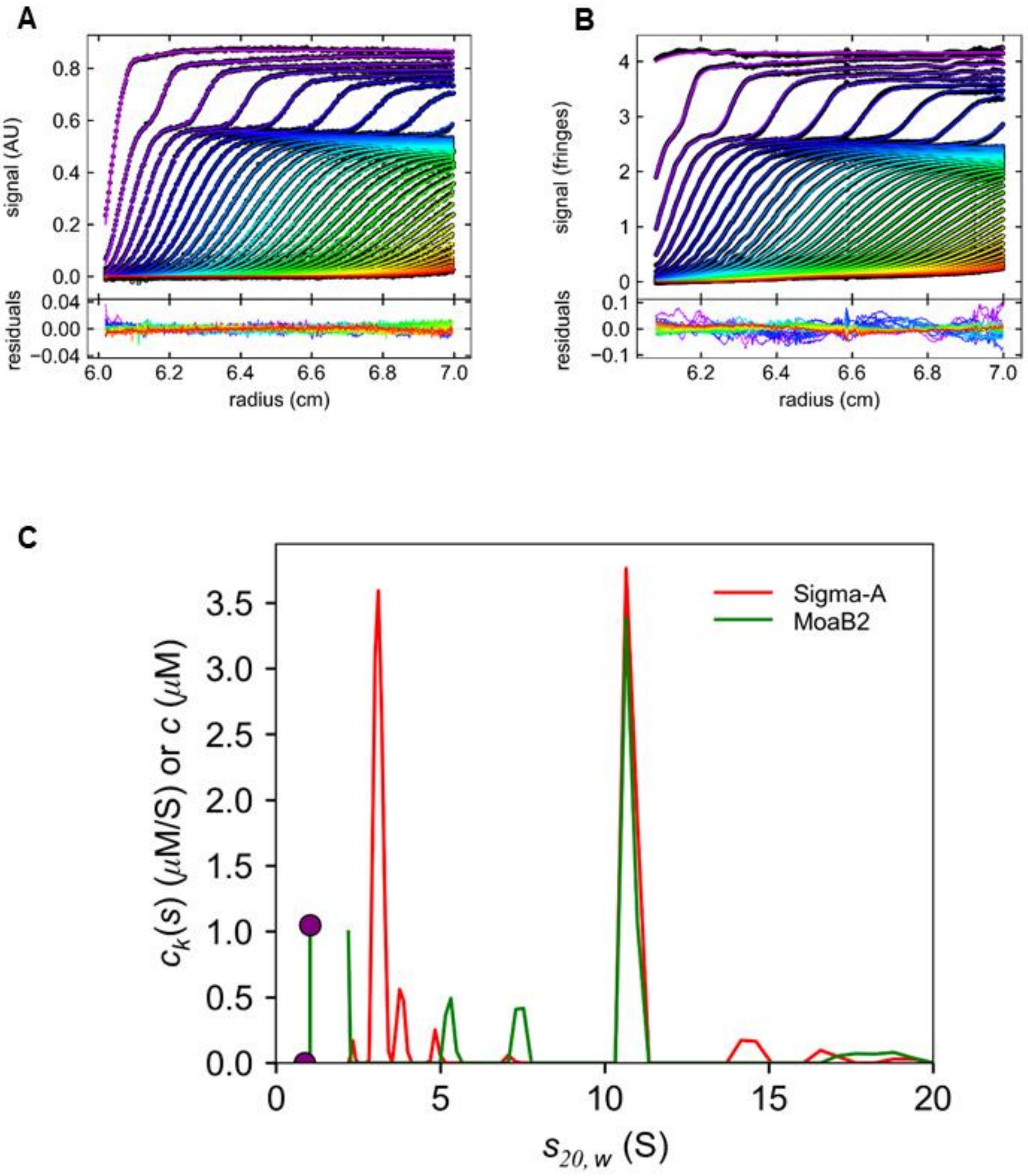

Supplementary Figure S4: Multi-signal sedimentation velocity (MSSV) analysis of the mixture of 19.4 μM σ^A and 5.8 μM MoaB2.

The multi-signal sedimentation velocity experiment was performed at 20 °C and 48,000 rpm using absorbance (280 nm) and interference detection.

A. Global fitting of absorbance data. For simplicity, every 3rd scan is shown. The residual graphs show the goodness of the fit.

B. Global fitting of interference data. For simplicity, every 3rd scan is shown. The residual graphs show the goodness of the fit.

C. $c_k(s)$ distributions of σ^A and MoaB2 after spectral decomposition. Integration of the ~10 S complex peak yields the approximately equimolar concentrations of both cosedimenting components (5.62 μM σ^A and 4.45 μM MoaB2). Please note that buffer components in the MoaB2 sample were modelled as discrete components and are indicated with purple circles.

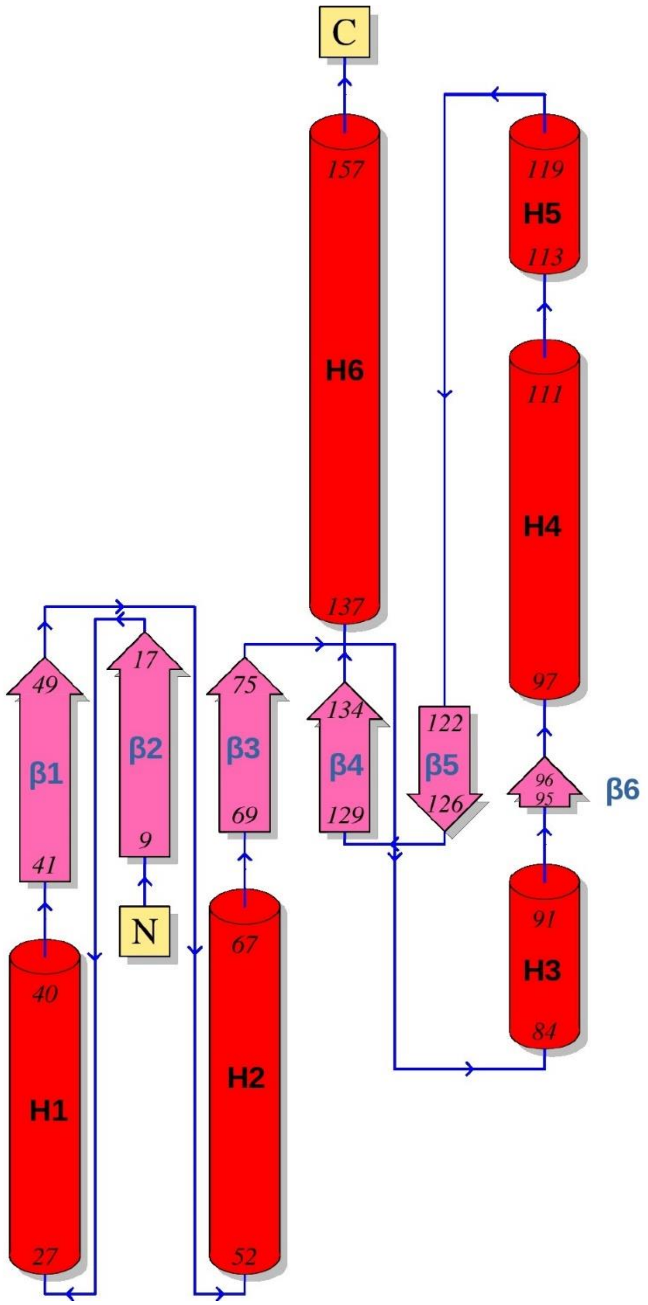

Supplementary Figure S5: Topology diagram of *M. smegmatis* MoaB2 crystal structure created by the PDBsum tool.

The diagram represents how the secondary structure elements (β-strands and α-helices) are organized and how these elements are linked. Red cylinders represent α-helices. Each large arrow indicates a single β-strand. The N- to C-terminal direction is indicated by thin arrows. Numbers indicate amino acids.

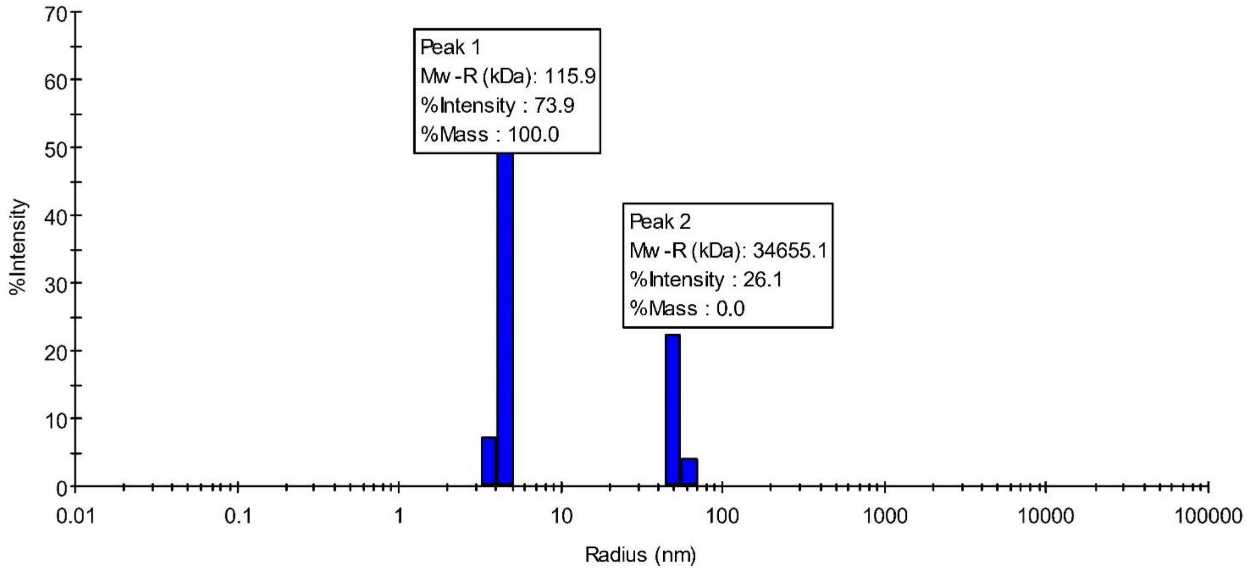

Supplementary Figure S6: Dynamic light scattering (DLS) data of *M. smegmatis* MoaB2.

The graph shows the particle size distribution, mean size values for each peak and corresponding scattering intensity percentages. The data was recorded, and the image was generated using DelsaMAX Core.

The DLS data show that the protein is present as a hexamer in solution (theoretical molecular mass 107 kDa, the major peak in the obtained distribution corresponds to 116 kDa).

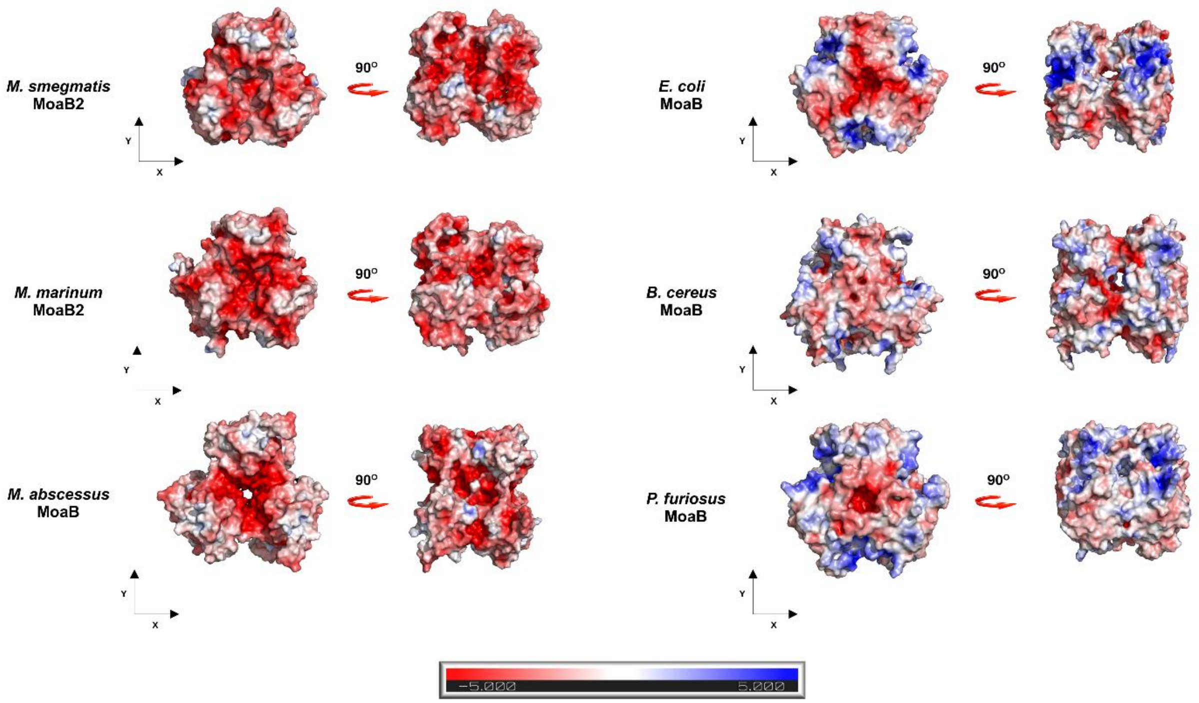

Supplementary Figure S7: Electrostatic potential distribution on the surface of MoaB and MoaB2 hexamers from different bacterial species.

Images for *M. smegmatis* (PDB id: 8byr), *M. marinum* (PDB id: 3rfq), *M. abscessus* (PDB id: 3tcr), *E. coli* (PDB id: 1mkz), *B. cereus* (PDB id: 1y5e) and *P. furiosus* (PDB id: 4lhb) were generated with the APBS Pymol plugin. The molecules are color-coded from red (negative potential, −5 k_BT/e, kB Boltzmann constant, T is temperature and e charge of electron) to blue (positive potential, +5 k_BT/e).

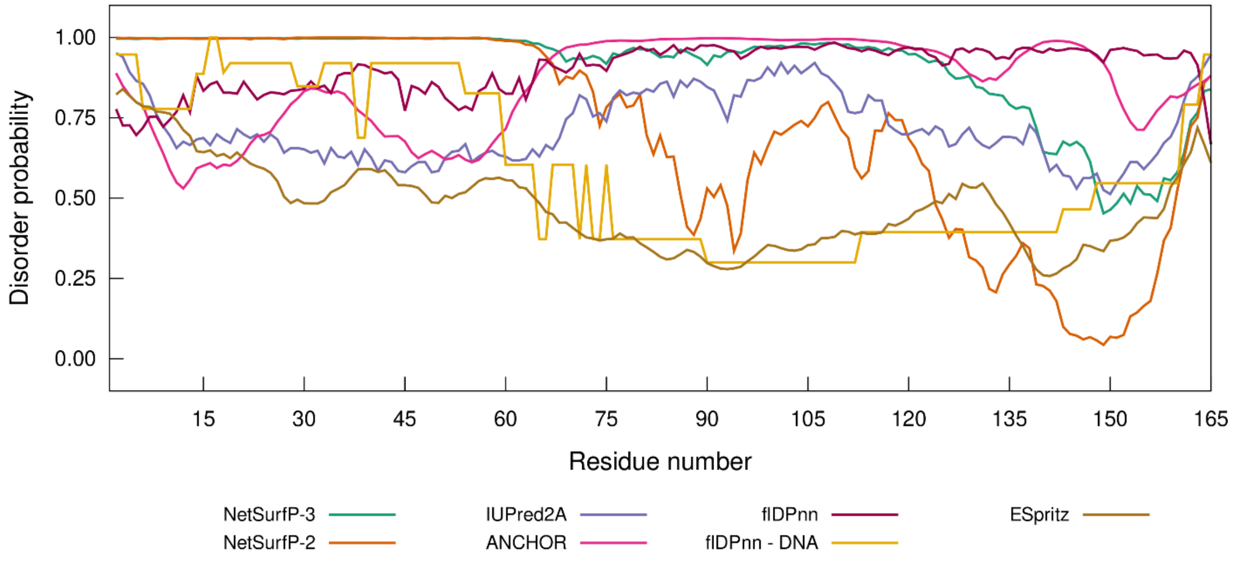

Supplementary Figure S8: Domain $\sigma^A{}_N$ of *M. smegmatis* σ^A is disordered.

Prediction of disorder from the sequence of *M. smegmatis* $\sigma^A{}_N$ using the following predictors: NetSurfP-3.0, green (1); NetSurfP-2.0, orange (2); IUPred2A, purple (3); ANCHOR, magenta (4); flDPnn, maroon (5), flDPnn – DNA binding propensity, yellow (5); ESpritz, brown (6). The predictors show high overall propensity towards disordered state of the $\sigma^A{}_N$, complementing the findings from NMR and circular dichroism spectroscopy.

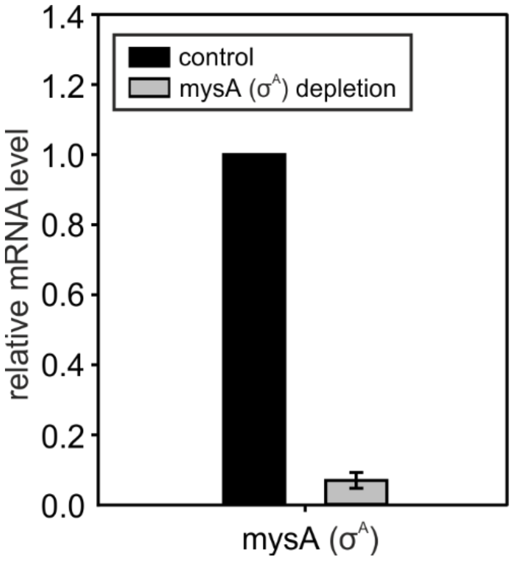

Supplementary Figure S9: Relative mysA (σ^A) mRNA level.

Relative quantitation of mysA (σ^A) mRNA in the σ^A CRISPR depletion strain (LK2203) (grey bar) compared to the control strain (LK2261; black bar, set as 1) determined by RT-qPCR. The mRNA level was normalized to an external spike (RNA control introduced during the RNA extraction protocol). The bar shows the average from three independent experiments and error bars show ± StDev.

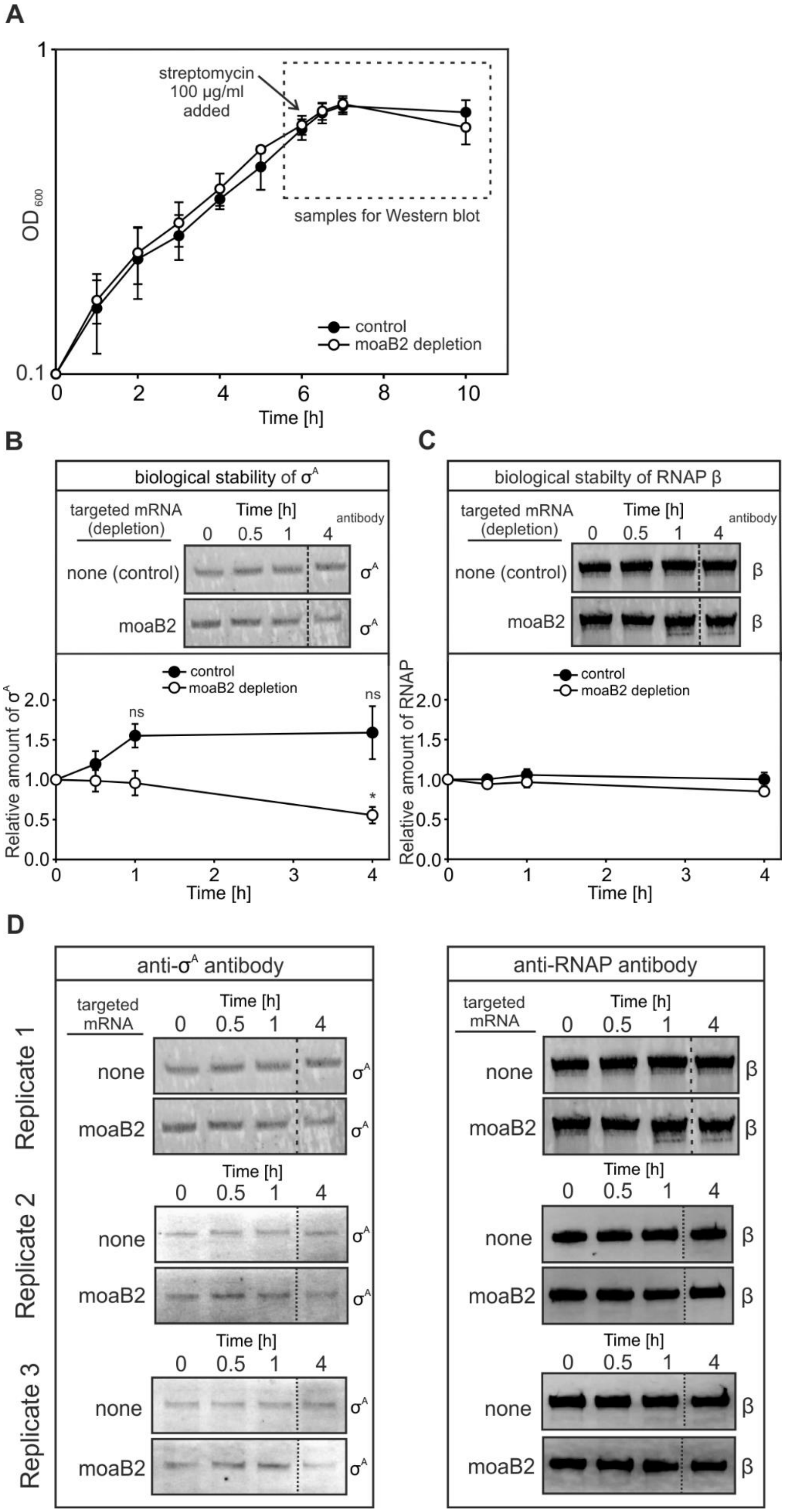
A
streptomycin 100 µg/ml added
samples for Western blot
OD 600
Time [h]
control
moaB2 depletion
B
biological stability of σA
targeted mRNA (depletion)
Time [h]
antibody
none (control)
moaB2
Relative amount of σA
ns
ns
*
C
biological stabilty of RNAP β
Relative amount of RNAP
D
anti-σA antibody
anti-RNAP antibody
Replicate 1
Replicate 2
Replicate 3
targeted mRNA
none
moaB2

Supplementary Figure S10: *M. smegmatis* MoaB2 affects stability of σ^A (previous page).

A. Growth curves (OD_{600}) of control strain (*M. smegmatis* strain containing non-targeting control sgRNA; LK2261) and moaB2 depletion strain (strain containing moaB2 targeting sgRNA; LK2263). Time (6h) of the addition of streptomycin (100 μg/ml) is indicated with an arrow. Dotted rectangle indicates 4 time points from which aliquots were taken for Western blot analysis. The data points show averages from three independent experiments and the error bars indicate ± StDev.

B. The relative level of σ^A and β subunit of RNAP (**C**) determined by Western blotting with σ^A or β antibody (**C**) before (time 0) and after the addition of streptomycin (100 μg/ml). Equal amounts of total protein were loaded in each lane. The upper panels show representative primary data. Control: *M. smegmatis* strain containing non-targeting control sgRNA (LK2261); moaB2, strain containing moaB2 targeting sgRNA (LK2263). The primary data come from one blot that was electronically assembled; the dotted lines indicate the points of assembly. The bottom panels show quantitation of the data. The relative level of σ^A or β subunit of RNAP (**C**) at time 0 was set as 1. The data points show averages from three independent experiments and the error bars indicate ± StDev. Decrease of σ^A in the *moaB2* depletion strain (LK2263) marked with asterisk (*) was statistically significant (p-value<0.05, t-test). Increase of σ^A in the control strain (LK2261) marked with 'ns' was not statistically significant (p-value>0.05, t-test).

D. The relative levels of σ^A and β subunit of RNAP were determined by Western blotting with σ^A or β antibodies, respectively, before (time 0) and after the addition of streptomycin (100 μg/ml). Equal amounts of total protein were loaded into each lane. Panels show primary data from three independent experiments. "None" served as a control, *M. smegmatis* strain containing non-targeting control sgRNA (LK2261); moaB2, strain containing moaB2 targeting sgRNA (LK2263). The primary data for each experiment come from one blot that was electronically assembled; the dotted lines indicate the points of assembly.

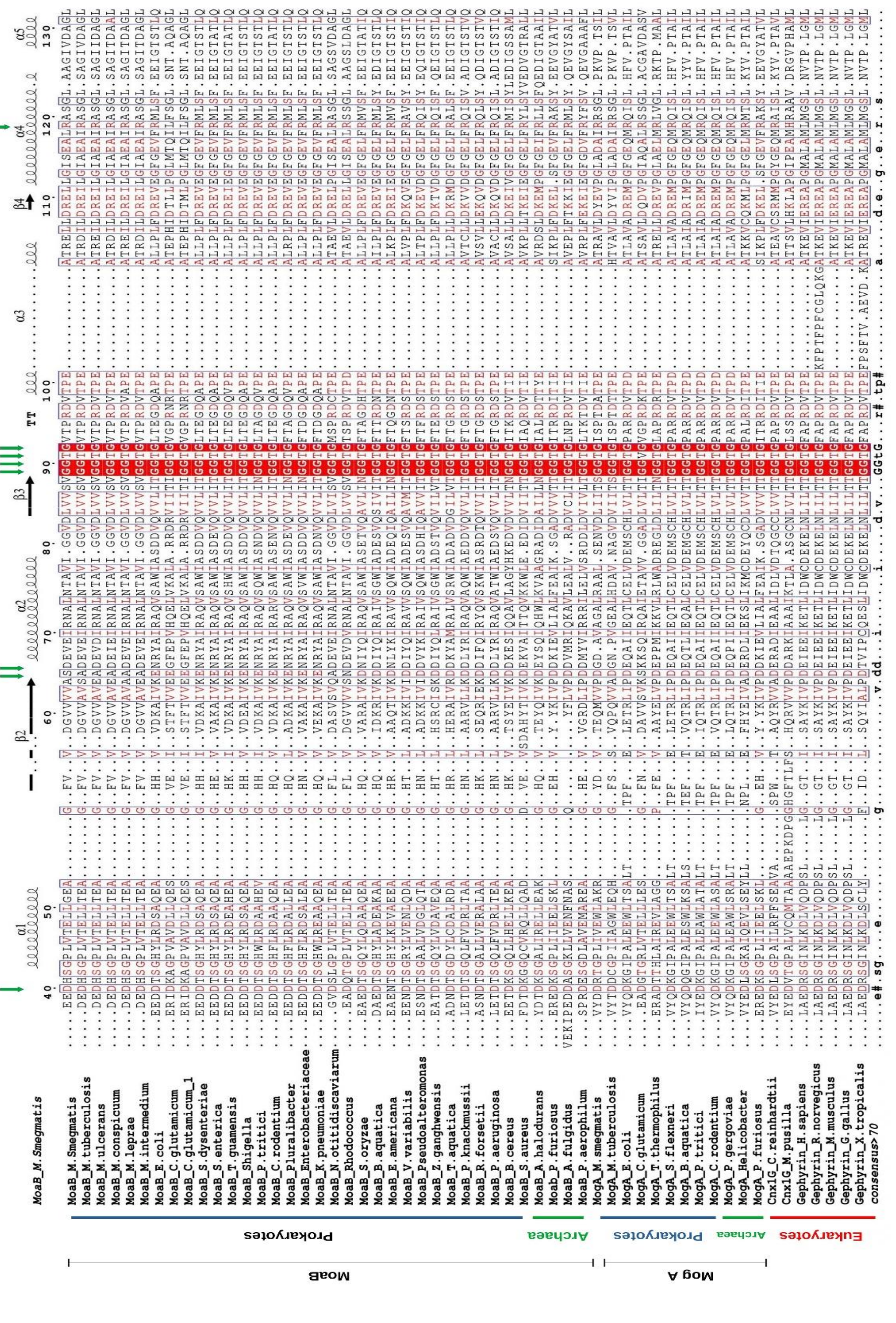
MoaB_M. Smegmatis
MoaB
Mog A
Prokaryotes
Archaea
Eukaryotes

Supplementary Figure S11: Sequence alignment of *M. smegmatis* MoaB2 with prokaryotic, archaeal, and eukaryotic homologs (previous page).

Residues are colored based on their conservation using the ESPript 3.0 server (7). Consensus sequence is shown at the bottom (capital letters represent strict conservation and small letters represent high conservation). Above the sequence alignment is the secondary structure of *M. smegmatis* MoaB2 (PDB id: 8byr). Green arrows correspond to the active site residues of the enzyme family capable of adenylation in the Molybdenum adenylating enzyme subfamily. The third green arrow from the left-hand side indicates the catalytically important residue discussed in the text (corresponding to *M. smegmatis* Ser51).

The alignment was performed with Tcoffee (8). MoaB2 shares a high level of sequence homology not only with bacterial MoaB and MogA proteins but also with the other members of the MPT adenyltransferase protein family. It has a high sequence identity of ~82% with MoaB from other mycobacterial species, 30-37% with MoaBs from other bacteria, 40% sequence identity with MogA in mycobacterial species, 29-35% with MogA in other bacteria, and 29-37% with plant Cnx1G and GephG.

pJET_Plat RNA transcript

GGGAGAGCGGCCGCAUGGGCAAGAGUUACACAGCGUGGAGGACCAACUCCCAG
GCACUCGGCCUGGGCAGACACAAUUAUUGUCGGAAUCCAGAUGGUGAUGCCAGA
CCUUGGUGCCAUGUGAUGAAGGACCGAAAGCUGACGUGGGAAUACUGUGACAU
GUCCCCAUGCUCCACCUGUGGCCUGAGGCAGUACAAACGGCCUCAGUUUAGAAU
UAAAGGAGGACUCUACACAGACAUCACCUCACACCCUUGGCAGGCUGCCAUCUU
UGUCAAGAACAAGAGGUCUCCUGGAGAGAGAUUCCUUUGUGGAGGGGUGCUGA
UCAGUUCCUGCUGGGUGCUGUCAGCUGCCCACUGCUUUCUAGAGAGGUUCCCC
CCCAAUCAUCUUAAAGUGGUCUUGGGCAGAACAUACAGGGUGGUCCCCGGAGAG
GAAGAACAGACAUUUGAGAUUGAAAAAUACAUAGUCCAUGAGGAAUUUGAUGACG
AUACUUAUGACAACGACAUCGCAUUACUGCAGCUGAGGUCACAGUCCAAGCAAU
GUGCCCAAGAGAGCAGCUCUGUUGGCACUGCCUGCCUCCCUGACCCCAACCUG
CAGCUCCCUGACUGGACAGAGUGUGAGCUUUCUGGCUACGGCAAGCAUGAGGC
AUCGUCUCCAUUCUUCUCUGCGGCCGCAAUCUUUCUAGAAGAUCUCCUACAAUA
UUCUCAGCUGCCAUGGAAAAU

Supplementary Figure S12: RNA sequence of the Plat fragment (718 nt) used as the RNA spike for RT-qRNA quantitation.

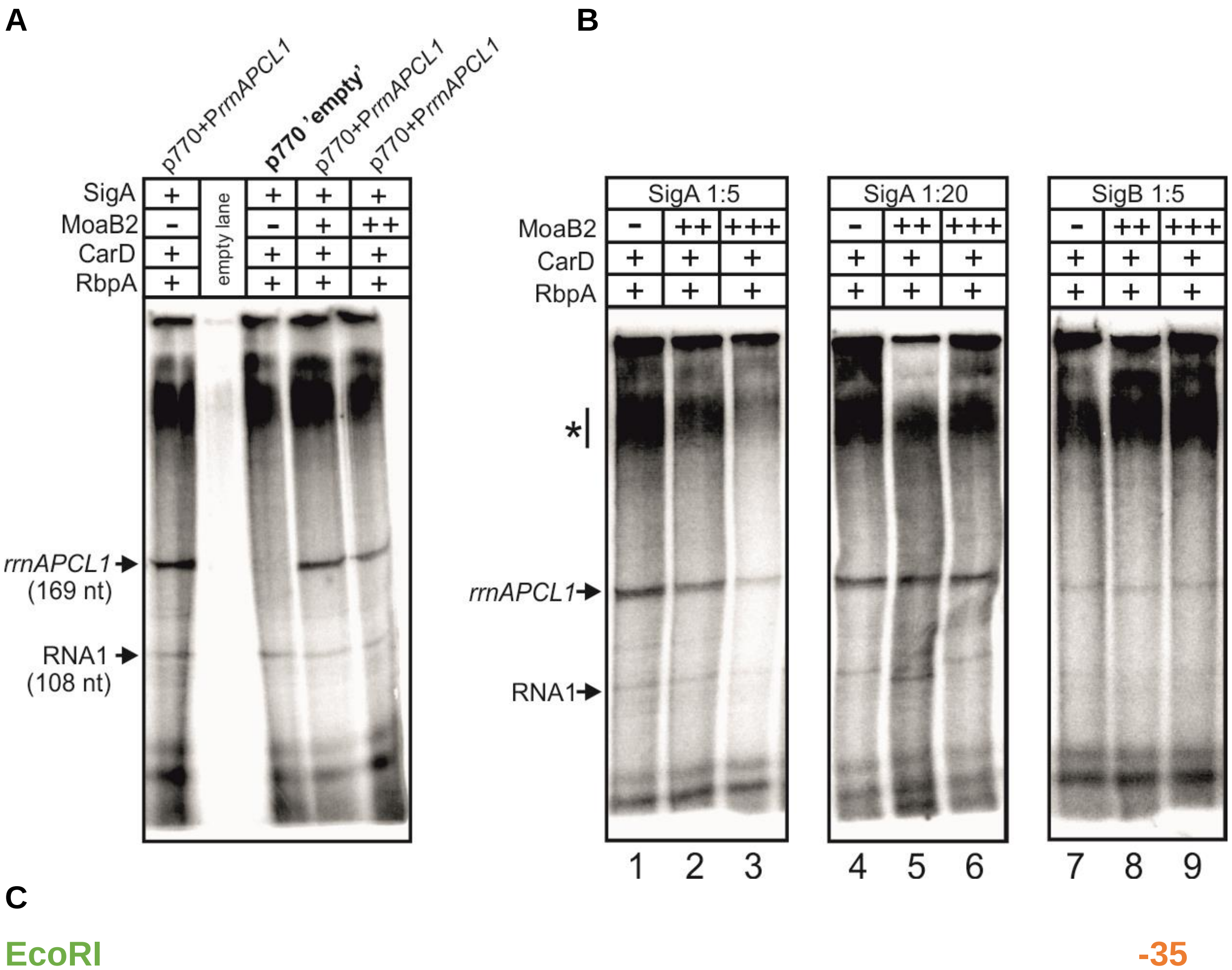

C

EcoRI **-35**
CTTAAGAATTCCGCGGAGCGGAGAAAACAACCCGGTCCAAGCGAC**TTGACA**

-10 **+1**
AGCCAGACAAAGCAGTAT**TAAGCT**GGCAGG**G**TTGCCCCAAAACGGGGCG

HindIII
AAGCTTGGGTCCCACCTGACCCCATGCCGAACTCAGAAGTGAAACGCCGT

AGCGCCGATGGTAGTGTGGGGTCTCCCCATGCGAGAGTAGGGAACTGCC

Stem Loop **Stem** T-region
AGGCATCAAATAAAAC**GAAAGGCTCA**GTCGAAAGA**CTGGGCCTTTC**G**TTTT**

Supplementary Figure S13: 7% polyacrylamide (PAA) gels of multiple round *in vitro* transcriptions from the p770 vector and respective promoter and transcript sequence used in *in vitro* transcription experiments.

A. Representative 7% PAA gel showing transcripts from a plasmid with a cloned P*rrnAPCL1* promoter (LK1548) versus transcripts from the p770 plasmid without any cloned promoter (LK2385). Transcription was carried by reconstituting σ^A +/- MoaB2 first

and then adding RNAP, CarD and RbpA. Two main transcripts are transcribed from the p770 plasmid containing the P*rrnAPCL1* promoter (9): 169 nt long *rrnAPCL1* and 108 nt long RNA1 (regulation of replication). The RNA products are marked and described on the left side of the gel. Concentrations and relative amounts of proteins used in this assay are the same as in **Fig. 8**: RNAP 0.06 μM, SigA 0.3 μM, MoaB2 0.3 μM or 1.8 μM, CarD 0.3 μM and RbpA 0.3 μM (for details see Mat&Met). The "empty" p770 plasmid template (without any cloned promoter) was used as a marker to demonstrate the identity of the P*rrnAPCL1* transcript.

B. Full images of 7% polyacrylamide gels from **Fig. 8**. The asterisk (*) marks nonspecific transcripts originating by readthrough of the terminator or arises due to spurious transcription from the plasmid (10).

Multiple-round transcriptions were performed with RNAP (LK1853) reconstituted with σ^A (LK2832) at the 1:5 and 1:20 ratios or σ^B (LK1248) at the 1:5 ratio in the absence or presence of increasing amounts of MoaB2 (LK2936) and at the presence of CarD (LK3209) and RbpA (LK3210) (indicated above the graph). Lane numbers are indicated below each gel. As promoter, the *M. smegmatis* rRNA promoter P*rrnAPCL1* was used in all panels (LK1548). The RNA products are marked and described on the left side of the panel by arrows.

C. Sequence of cloned DNA fragment containing the *M. smegmatis* rRNA promoter P*rrnAPCL1* via EcoRI and HindIII restriction sites into the p770 plasmid. Respective restriction sites are indicated. The -10 and -35 hexamers and transcription start site (+1G) are indicated. The 169 nucleotides long transcript (highlighted by grey) terminates downstream of the HindIII restriction site on the Rho-independent intrinsic *E.coli rrnB* terminator that was inserted into p770 during its construction (11).

Supplementary Table S2: Data collection and refinement statistics for the crystal structure of *M. smegmatis* MoaB2.

Values in parentheses are for the highest resolution shell.

**PDB ID**	8BYR
**Space group**	$P2_12_12_1$
X-ray source	PETRA III, EMBL c/o DESY BEAMLINE P14 (MX2)
Wavelength (Å)	0.9762
Data-collection temperature (K)	100
**Unit cell**	
a, b, c (Å)	91.3, 105.6, 106.9
α, β, γ (°)	90.0, 90.0, 90.0
**Data collection**	
Resolution range (Å)	47.69 - 2.53 (2.53-2.64)
Wavelength (Å)	0.9762
Total number of reflections	470613 (56564)
Number of unique reflections	35068 (4206)
Completeness (%)	100 (100)
Mean I/σ (I)	15.0 (3.4)
R_{merge} (%)	3.8 (83.6)
R_{pim} (%)	1.1 (24.2)
R_{meas} (%)	4.0 (90.2)
$CC_{1/2}$	0.999 (0.872)
Mosaicity (°)	0.47
**Refinement**	
Resolution range (Å)	47.69 - 2.53 (2.53-2.64)
Number of reflections used	35068
R_{work}	0.192
R_{free}	0.260
Cruickshank's DPI (Å)	0.4475
Root mean square deviation from ideal values	
bonds lengths (Å)	0.0074

bonds angles (°)	1.5021
Number of molecules in AU	6
Solvent content (%)	49.59
Number of non-H protein atoms	6361
Number of water molecules	39
Wilson B-factor (Å^2)	47.7
Average B-factor (Å^2)	51.0
**Ramachandran plot (%)**	
in preferred regions	96.63
in allowed regions	4.36
in disallowed regions	0

Supplementary Table S4: List of assigned backbone amide chemical shifts obtained on $\sigma^{A}{}_{N}$ (1-160).

Residue	^{15}N / ppm	^{1}H / ppm
S7	117.109	8.308
V14	120.743	8.133
T21	119.081	8.290
S34	116.788	8.266
G40	108.253	8.314
G41	108.544	8.186
K42	120.786	8.158
G56	110.279	8.455
T57	113.621	8.014
E62	120.416	8.751
D63	121.860	8.380
G64	108.852	8.201
V65	119.680	7.951
T66	118.271	8.240
V71	122.211	8.186
T72	118.114	8.281
G80	109.338	8.440
E81	120.354	8.193
V85	119.717	7.941
E86	124.431	8.454
T88	114.468	8.051
L92	123.050	8.148
D93	121.092	8.306
S97	116.040	8.140

T101	114.456	8.069
A102	127.232	8.222
V103	119.962	8.102
E104	124.819	8.480
A110	125.884	8.370
D111	119.723	8.325
A112	124.523	8.115
A113	122.794	8.206
T114	116.382	8.051
A116	124.825	8.372
V117	119.540	8.051
A122	126.595	8.402
A123	123.556	8.320
D124	119.162	8.230
I127	120.352	7.939
D128	124.589	8.356
S131	116.496	8.498
K135	121.699	8.071
A136	124.692	8.247
S137	115.004	8.182
G138	110.585	8.273
D139	120.186	8.097
F140	120.540	8.021
V141	123.133	7.829
W142	125.548	8.144
D143	122.676	8.192
S147	116.758	8.142

E148	123.635	8.541
A149	122.216	8.137
L150	119.721	7.802
R151	120.448	7.979
Q152	119.273	8.210
A153	123.001	7.942
R154	119.231	7.980
K155	121.949	8.067
A157	122.862	7.918
E158	119.097	8.124
L159	122.947	8.092
T160	119.911	7.663

Supplementary Table S5: List of strains and plasmids.

Strain or plasmid	Relevant characteristics	Source or reference
M. smegmatis		
LK3016	*M. smegmatis* mc^2 155, no FLAG	Laboratory strain
LK2678	*E. coli* Lemo21 (DE3) expression strain	New England Biolabs (NEB)
LK1321	*M. smegmatis* mc^2 155 containing pJV53	(12)
LK3207	σ^A-FLAG integrated into *M. smegmatis* mc^2 155 genome at the native locus	This work
LK2073	σ^A-FLAG integrated into pTetInt and transformed into *M. smegmatis* mc^2 155	(13)
LK1468	RNAP-FLAG (β-FLAG) was kindly provided by D. Schnappinger, Weill Cornell Medical College, New York, USA	(14)
LK2463	$\sigma^A_{\Delta N}$-FLAG cloned into pTetInt and transformed into *M. smegmatis* mc^2 155	This work
LK4207	$\sigma^A_{\Delta 60aaN}$-FLAG cloned into pTetInt and transformed into *M. smegmatis* mc^2 155	This work
LK2077	σ^B-FLAG cloned into pTetInt and transformed into *M. smegmatis* mc^2 155	This work
LK2157	σ^E-FLAG cloned into pTetInt and transformed into *M. smegmatis* mc^2 155	This work
LK2159	σ^F-FLAG cloned into pTetInt and transformed into *M. smegmatis* mc^2 155	This work
LK2161	σ^G-FLAG cloned into pTetInt and transformed into *M. smegmatis* mc^2 155	This work
LK2160	σ^H-FLAG cloned into pTetInt and transformed into *M. smegmatis* mc^2 155	This work
LK2261	CRISPR Cas9 negative control, non-targeting	(9)

	sgRNA	
LK2263	CRISPR Cas9 moaB2 knockdown	This work
LK2203	CRISPR Cas9 σ^A knockdown	This work
E. coli		
LK13	*E. coli* DH5α competent cells	Laboratory strain
LK625	*E. coli* BL21 (DE3) competent cells	Laboratory strain
LK1463	pUC18-hygromycine resistance, DH5α	Laboratory strain
LK2385	p770 without a cloned promoter, DH5α	(15)
LK1548	p770+P*rrnAPCL1*, DH5α	(13)
LK1740	σ^A-His, Lemo21 (DE3)	(13)
LK2844	pET28bMBP/σ^A, DH5α	This work
LK2832	His-MBP-σ^A, Lemo21 (DE3)	This work
LK2863	$\sigma^A_{N(1\text{-}160)}$-MBP-His, Lemo21 (DE3)	This work
LK2864	σ^A_N-MBP-His, Lemo21 (DE3)	This work
LK1853	RNAP-His, BL21 (DE3)	(16)
LK3210	RbpA-His, Lemo21 (DE3)	This work
LK2938	pET302/His-MoaB2, DH5α	This work
LK2936	His-MoaB2, Lemo21 (DE3)	This work
LK3679	pET302/His-CarD, DH5α	This work
LK3209	His-CarD, Lemo21 (DE3)	This work
LK4295	pET22b/His-σ^B, DH5α	This work
LK1248	His-σ^B, DE3	This work
LK1345	*B. subtilis* $\sigma^A_{1.1}$-His, BL21 (DE3)	(17)
LK2615	MoaB2-His, BL21 (DE3)	This work

Supplementary Table S6: List of oligonucleotides.

If one Comment section describes two primers, the first primer is in each case the Forward primer and the second primer the Reverse primer.

Primer number	Sequence (5'-3')	Comment
#5033	CGCCATATGCCCGAGGACGGCGTCACCGAC	Primers for $\sigma^{A}_{\Delta 60aaN}$-1xFLAG (*MSMEG_2758*) cloning into pTetInt
#5034	CCCAACGTTCTACTTGTCGTCGTCGTCCTTGTAGTCGTCCAGGTAGTCGCGCAGCACCTG	
#2901	ATTCCATATGGCATCGGCCGACTCGGTTCGC	Primers for $\sigma^{A}_{\Delta N}$-3xFLAG (*MSMEG_2758*) cloning into pTetInt
#2340	TGTTAATTAACTACTTGTCGTCGTCGTCCTTGTAGTCCAGGTAGTCGCGCAGCAC	
#2337	CTTCATATGGCAAATGCCACCACAAGCCGC	Primers for σ^{B}-3xFLAG (*MSMEG_2752*) cloning into pTetInt
#2392	CCGTCGTGGTCCTTGTAGTCGCTCGCGTAGGAGCGGAGGCG	
#2343	CTTCATATGGAACACGACGACCGTCGCGCC	Primers for σ^{E}-3xFLAG (*MSMEG_5027*) cloning into pTetInt
#2395	CCGTCGTGGTCCTTGTAGTCGGCGGACTGTGCGGTTTCCGACGAAT	
#2345	CTTCATATGACGTCGGAATACGCAGACGTT	Primers for σ^{F}-3xFLAG (*MSMEG_1804*) cloning into pTetInt
#2396	CCGTCGTGGTCCTTGTAGTCCTGCAGCTGGTCGCGCAGCCGC	
#2374	CTTCATATGTCGGTCATCCTGCGTAAGCTC	Primers for σ^{G}-3xFLAG (*MSMEG_0219*) cloning into pTetInt
#2397	CCGTCGTGGTCCTTGTAGTCCAGCGTTTCGGGCAGCCCGAA	

#2349	CTTCATATGTTTCCCACGATGACTGACGTC	Primers for σ^H-3xFLAG (*MSMEG_1914*) cloning into pTetInt
#2398	CCGTCGTGGTCCTTGTAGTCTGACGTCACCTCCTCGGGTTC	
#3295	CGTTGTAAAACGACGGCCAGTGCCAAGCTTACGCCACGTGGTGGATCC	Primers for cloning of σ^A-FLAG LA into pUC18
#3296	GTTAACCTGCAGCTACTTGTCGTCGTCGTCCTTG	
#3297	CGACGACAAGTAGCTGCAGGTTAACGAAATCAATC	Primers for cloning of σ^A-FLAG HYG into pUC18
#3306	CTCAGGAGTTCTTTCTAGAGAAGTTATCCCGGG	
#3307	AACTTCTCTAGAAAGAACTCCTGAGATGACGGC	Primers for cloning of σ^A-FLAG RA into pUC18
#3308	TACGAATTCGAGCTCGGTACCCGGGGATCCTCCGTATCGACCCCGCCAAAC	
#2385	CGTAAGCTTCTACTTGTCGTCGTCGTCCTTGTACTCGATGTCGTGGTCCTTGTAGTCGCCGTCGTGGTCCTTGTAGTC	3x FLAG tag with HindIII restriction site
#3632	CGAGGAAAACCTGTACTTCCAGGGTATGGAACAGCCAGGGGAGTTG	Primers for cloning of MoaB2 (*MSMEG_5485*) into pET302 (TEV cleavage)
#3633	CTTTCGGGCTTTGTTAGCAGCCGGATCCTTAGATCTCCAAGCTTGATAG	
#TK1	CAGGAGAACCTGTACTTCCAGGGCATGGCAGCGACAAAGGCAAG	Primers for cloning of σ^A (*MSMEG_2758*) into pET28-MBP-TEV (TEV cleavage)
#TK2	TGCCCTGGAAGTACAGGTTTTCTTCTCGAGCTAGTCCAGGTAGTCGC	
#3775	CGAGGAAAACCTGTACTTCCAGGGTATGATTTTTAAGGTCGGAGAC	Primers for cloning of CarD (*MSMEG_6077*) into pET302 (TEV
#3776	CTTTCGGGCTTTGTTAGCAGCCGGATC	

	CTTAGGACGCGGCGGCCAAAACC	cleavage)
#3189	ATTCCATATGGAACAGCCAGGGGAGTT	Primers for cloning of MoaB2 (*MSMEG_5485*) into pET22b
#2472	CCGCTCGAGGATCTCCAAGCTTGATAGCTG	
#1153	GGAATTCCATATGATGGCAAATGCCACCACA	Primers for cloning of σ^B (*MSMEG_2752*) into pET22b
#1154	CCGCTCGAGGCTCGCGTAGGAGCGGAG	
#1474	AATTCCGCGGAGCGGAGAAAACAACCCGGTCCAAGCGACTTGACAAGCCAGACAAAGCAGTATTAAGCTGGCAGGGTTGCCCCAAAACGGGGCGA	Primers for cloning of pAPCL1 cloning into p770
#1475	AGCTTCGCCCCGTTTTGGGGCAACCCTGCCAGCTTAATACTGCTTTGTCTGGCTTGTCAAGTCGCTTGGACCGGGTTGTTTTCTCCGCTCCGCGG	
#2455	GGGAGTGGGCATGGGCATGCTTCA	Primers for cloning of σ^A CRISPR Cas9 strain
#2456	AAACTGAAGCATGCCCATGCCCAC	
#2484	GGGAGACCACGAACCCGGCCTCAC	Primers for cloning of *moaB2* CRISPR Cas9 strain
#2485	AAACGTGAGGCCGGGTTCGTGGTC	
#2476	GCGTCGATCTGGTGGTGT	Primers for *moaB2* (*MSMEG_5485*) used in RT-qPCR
#2477	GCTCACGATCCAGCAACTCT	
#987	CCAAGGGCTACAAGTTCTCG	Primers for σ^A (*MSMEG_2758*, *mysA*) used in RT-qPCR (9)
#988	CTTGTTGATCACCTCGACCA	
#989	CGACGAGATCTGGAACACCT	Primers for *rpoC* (*MSMEG_1368*) used
#990	GGTGAAGTACTCGCCGTAGC	

		in RT-qPCR (9)
#3542	TTACTGCAGCTGAGGTCACA	Primers for Plat_spike used in RT-qPCR
#3543	AGCTCACACTCTGTCCAGTC	

Supplementary References

1. Klausen MS, Jespersen MC, Nielsen H, Jensen KK, Jurtz VI, Sønderby CK, Sommer MOA, Winther O, Nielsen M, Petersen B, Marcatili P. 2019. NetSurfP-2.0: Improved prediction of protein structural features by integrated deep learning. Proteins 87:520–527.

2. Høie MH, Kiehl EN, Petersen B, Nielsen M, Winther O, Nielsen H, Hallgren J, Marcatili P. 2022. NetSurfP-3.0: accurate and fast prediction of protein structural features by protein language models and deep learning. Nucleic Acids Res 50:W510–W515.

3. Mészáros B, Erdös G, Dosztányi Z. 2018. IUPred2A: context-dependent prediction of protein disorder as a function of redox state and protein binding. Nucleic Acids Res 46:W329–W337.

4. Dosztányi Z, Mészáros B, Simon I. 2009. ANCHOR: web server for predicting protein binding regions in disordered proteins. Bioinformatics 25:2745–2746.

5. Hu G, Katuwawala A, Wang K, Wu Z, Ghadermarzi S, Gao J, Kurgan L. 2021. flDPnn: Accurate intrinsic disorder prediction with putative propensities of disorder functions. Nat Commun 12.

6. Chai H, Gu Q, Hughes J, Robertson DL. 2022. In silico prediction of HIV-1-host molecular interactions and their directionality. PLoS Comput Biol 18:e1009720.

7. Robert X, Gouet P. 2014. Deciphering key features in protein structures with the new ENDscript server. Nucleic Acids Res 42:W320–W324.

8. Notredame C, Higgins DG, Heringa J. 2000. T-coffee: a novel method for fast and accurate multiple sequence alignment. J Mol Biol 302:205–217.

9. Šiková M, Janoušková M, Ramaniuk O, Páleníková P, Pospíšil J, Bartl P, Suder A, Pajer P, Kubičková P, Pavliš O, Hradilová M, Vítovská D, Šanderová H, Převorovský M, Hnilicová J, Krásný L. 2019. Ms1 RNA increases the amount of RNA polymerase in Mycobacterium smegmatis. Mol Microbiol 111:354–372.

10. Ross W, Gourse RL. 2009. Analysis of RNA polymerase-promoter complex formation. Methods 47:13–24.

11. Ross W, Thompson JF, Newlands JT, Gourse RL. 1990. E.coli Fis protein activates ribosomal RNA transcription in vitro and in vivo. EMBO J 9:3733–3742.

12. van Kessel JC, Hatfull GF. 2006. Recombineering in Mycobacterium tuberculosis. Nature Methods 2006 4:2 4:147–152.

13. Kouba T, Koval' T, Sudzinová P, Pospíšil J, Brezovská B, Hnilicová J, Šanderová H, Janoušková M, Šiková M, Halada P, Sýkora M, Barvík I, Nováček J, Trundová M, Dušková J, Skálová T, Chon UrR, Murakami KS, Dohnálek J, Krásný L. 2020. Mycobacterial HelD is a nucleic acids-clearing factor for RNA polymerase. Nature Communications 2020 11:1 11:1–13.

14. Abdelkareem M, Saint-André C, Takacs M, Papai G, Crucifix C, Guo X, Ortiz J, Weixlbaumer A. 2019. Structural Basis of Transcription: RNA Polymerase Backtracking and Its Reactivation. Mol Cell 75:298-309.e4.

15. Ross W, Thompson JF, Newlands JT, Gourse RL. 1990. E.coli Fis protein activates ribosomal RNA transcription in vitro and in vivo. EMBO J 9:3733–3742.

16. Molodtsov V, Sineva E, Zhang L, Huang X, Cashel M, Ades SE, Murakami KS. 2018. Allosteric Effector ppGpp Potentiates the Inhibition of Transcript Initiation by DksA. Mol Cell 69:828-839.e5.

17. Zachrdla M, Padrta P, Rabatinová A, Šanderová H, Barvík I, Krásný L, Žídek L. 2017. Solution structure of domain 1.1 of the σA factor from Bacillus subtilis is preformed for binding to the RNA polymerase core. J Biol Chem 292:11610–11617.
